# Presence, flow, and narrative absorption: an interdisciplinary theoretical exploration with a new spatiotemporal integrated model based on predictive processing

**DOI:** 10.12688/openreseurope.13193.1

**Published:** 2021-03-26

**Authors:** Federico Pianzola, Giuseppe Riva, Karin Kukkonen, Fabrizia Mantovani

**Affiliations:** 1 Department of Human Sciences for Education "R. Massa", University of Milan Bicocca, Milan, Italy; 2 School of Media, Arts and Science, Sogang University, Seoul, South Korea; 3 Department of Psychology, Universita Cattolica del Sacro Cuore, Milan, Italy; 4 Applied Technology for Neuro-Psychology Lab, Istituto Auxologico Italiano, Milan, Italy; 5 Department of Literature, Area Studies and European Language, University of Oslo, Oslo, Norway

**Keywords:** Presence, flow, narrative absorption, immersion, predictive processing, active inference, book problem, paradox of fiction

## Abstract

Presence, flow, narrative absorption, immersion, transportation, and similar subjective phenomena are studied in many different disciplines, mostly in relation to mediated experiences (books, film, VR, games). Moreover, since real, virtual, or fictional agents are often involved, concepts like identification and state empathy are often linked to engaging media use. Based on a scoping review which identified similarities in the wording of various questionnaire items conceived to measure different phenomena, we categorize items into the most relevant psychological aspects, and use this categorization to propose an interdisciplinary systematization. Then, based on a framework of embodied predictive processing, we present a new cognitive model of presence-related phenomena for mediated and non-mediated experiences, integrating spatial and temporal aspects and also considering the role of fiction and media design. Key processes described within the model are: selective attention, enactment of intentions, and interoception. We claim that presence is the state of perceived successful agency of an embodied mind able to correctly enact its predictions. The difference between real-life and simulated experiences (“book problem,” “paradox of fiction”) lays in the different precision weighing of exteroceptive and interoceptive signals.

## 1. Introduction

Many different disciplines have studied how technology mediates experience, in particular cultural and aesthetic ones, like reading a book, watching a film, or playing a game in virtual reality. Over the years, the lack of cooperation across disciplinary boundaries has led to a research landscape characterized by related and overlapping concepts, like *presence* ( Lombard *et al.*, 2015), *flow* ( Csikszentmihalyi, 1990; Harmat *et al.*, 2016), and *narrative absorption* ( Hakemulder *et al.*, 2017). Some of these concepts, have been primarily introduced for activities not necessarily related to the mediation of cultural and technological artefacts (e.g. flow; Csikszentmihalyi, 1990); some others have been introduced in relation to a specific technology (e.g. presence in virtual reality; Sheridan, 1992), and have been later acknowledged to be broader psychological phenomena not necessarily linked to the experience of a medium ( Coelho *et al.*, 2012; Mantovani & Riva, 1999). After thirty years of empirical research on the experiences described by such concepts, we attempt here to organize knowledge from various fields, ranging from neuropsychology, cognitive narratology, empirical aesthetics, communication studies, and media psychology. Our main goal is to explain what cognitive and affective processes are activated when we feel “present” or “immersed” in a simulated world (virtual, imagined, or fictional). This is sometimes referred to – with different meanings – as the “book problem” ( Biocca, 2003) or the “paradox of fiction” ( Sperduti *et al.*, 2017b; Walton, 1978). In order to provide a satisfactory explanation, we first clarify the relationships between similar phenomena labelled in different ways. Then, we present a new integrated model of presence and related concepts, based on predictive embodied cognition ( Clark, 2016; Friston, 2012; Hohwy, 2013; Ramstead *et al.*, 2020). We discuss its various aspects in relation to both mediated (virtual, imagined, and fictional) and unmediated (“real-world”) experiences.

The common interest of the many disciplines that tackle the topic of presence revolves around two issues that can be synthetized as:

- *presence*: how do we fit in the (real, virtual, or fictional) world?
- *social presence*: how do we relate and respond to (real, virtual, or fictional) others’ intentions?

Moreover, in many cases an additional goal is to describe experiences that grasp the attention for a relatively long time (narrative absorption) and that are considered to be particularly rewarding (flow). Therefore, beside *space* and *others*, two additional important elements that we discuss are *attention* and *time*. Many people often refer to these kinds of experiences with metaphors such as “engaging,” “immersive,” or “absorbing,” but our theoretical investigation goes beyond the various linguistic expressions and investigates cognitive and affective communalities and diversities among concepts and experiences.

Predictive processing (PP) is the framework of our model, grounded in a 4E conception of the mind: embodied, embedded in the social context, extended into the environment, and enactive ( Newen *et al.*, 2018; Varela *et al.*, 1991). Starting from this premise, we propose a model that integrates the concepts of *presence, social presence,* and *narrative absorption*, reconceptualizing them as spatiotemporal configurations of the subjective experience of an embodied cognitive agent. In brief, subjects are *present* where they are able to correctly and intuitively enact (i.e. without the involvement of reasoning) their implicit (predictive processing) and explicit (intentions) embodied predictions ( Kukkonen, 2020; Riva, 2018; Riva *et al.*, 2015) ( section 5.1).

In order to maintain a successful relation with the environment, organisms formulate hierarchical embodied predictions about their interaction with it on the basis of various multisensory stimuli ( Riva, 2018): exteroception, the perceptual information originating outside the body; interoception, the sense of the physiological and emotional condition of the body; proprioception, the sense of the relative position of body segments; and vestibular input, the sense of body motion. In the case of mediated experiences, multisensory stimuli affect the enactment of predictions in different ways with respect to physical/digital tools (e.g. ball/video game) and symbolic tools (e.g. narrative). The main difference is related to the *precision* (a measure of the reliability of prediction errors and their relevance for updating the model generating predictions) attributed to the different layers of embodied predictions ( Kukkonen, 2019a; Seth & Friston, 2016): predictions related to physical/digital tools and physical/digital objects attribute higher precision to exteroceptive and proprioceptive layers and/or vestibular information, according to the activity performed ( section 5.3). Instead, predictions related to symbolic tools attribute higher precision to interoceptive information ( section 5.4). This difference holds true also for the interaction with real, virtual, or fictional agents ( section 5.5). Moreover, self-related variables, too, like intentions or point of view (egocentric or allocentric), influence the precision of the different layers and the different relevance assigned to them ( section 5.2). Finally, in *narrative* the temporal unfolding of an experience, which is rooted in our body ( Di Lernia *et al.*, 2018; Richter & Ibáñez, 2021), makes interoceptive states ( Wittmann, 2009) much more relevant than in other cases. Temporality affects the enactment of intentions, consequently influencing the sense of presence ( section 5.6).

In synthesis, our model is characterized by the following aspects:

- It describes presence as a general psychological phenomenon related to different psychological functions: intentions and predictions, actual or simulated perception and action ( Riva *et al.*, 2015), attention oscillating between interoception and exteroception ( Kukkonen, 2019a).
- It is based on an embodied predictive processing model of the mind that extends the standard predictive processing scheme by incorporating interoceptive information and the perception of the physiological condition of the body ( Pezzulo, 2014; Seth *et al.*, 2012).
- Embodied predictions are framed in an evolutionary agentive account. On the one hand, they have a constraint in the agentive capacity of the subject; on the other hand, presence is used to minimize the level of free-energy through the correct prediction of exteroceptive/interoceptive inputs ( Friston & Stephan, 2007; Riva & Waterworth, 2014; Waterworth & Riva, 2014).
- It is valid for both mediated and non-mediated experiences, and we provide examples of real-world action, virtual reality (VR), literature, and video games.
- It does not multiplicate entities (e.g. real world, virtual world, mental imagery world, fictional world, text world) in order to account for the emergence of presence in relation to different situations.
- It describes a hierarchical organization of psychological functions characterized by feedback loops, explicitly showing the connection between presence and similar phenomena that have been studied under different names (narrative absorption, narrative engagement, transportation, immersion, flow).
- It considers how the narrative organization of information influences presence and is retroactively influenced by it.
- It considers the dynamics of attention when breaks in presence and narrative absorption occur.

By bringing together expertise from different disciplines, we show, among other things, how research in literary theory and empirical aesthetics can help to understand social presence outside fictional contexts (e.g. presenting the role of narrative elements in activating the enactment of characters’ consciousness), and how research in psychology and on VR can help to understand narrative absorption with stories and fictional characters. To help readers to follow this interdisciplinary dialogue, in Table 1 we provide a glossary of the main terms used in this article.

**Table 1.** A glossary of some of the terms used in this paper (alphabetic order).

Active inference	A specific version of PP according to which organisms minimize prediction errors by performing actions that confirm sensory predictions.
Attention	*Sensory selective attention* operates by selecting (automatically, *exogenous att.*; voluntarily, *endogenous att*.) certain stimuli over others. Cognitive and physical resources are allocated by *focused attention* based on the prevailing task demands. Depending on the kind of activity, these processes can require *sustained* *attention* in order to extend over a relatively long period of time.
Enactment	A model according to which cognition and emotions emerge from the continuous interaction between an acting organism and its environment.
Exteroception	Signals originating in the external environment and perceived through the senses.
Flow	A subjective-phenomenal state that can emerge while performing an activity, characterized by intense and focused concentration on the present moment and by perception of the activity as intrinsically rewarding. A crucial aspect for its emergence is the optimal balance between an agents’ skills and the challenge posed by the activity.
Free-energy principle	A formalization of the dynamics according to which organisms resist a natural tendency to disorder by remaining in non-equilibrium states that allows them to adapt to a constantly changing environment. This happens by self-limiting the number of physiological and sensory states in which an organism can be (free-energy minimization). Minimizing free-energy corresponds to explaining away prediction errors.
Immersion	A subjective-phenomenal state characterized by rich sensory stimuli (and often also perceived possibility of action) in a real, virtual, or imagined environment. Sensorimotor immersion is sometimes considered to be a prerequisite, or a constitutive part, of the sense of presence in mediated experiences.
Intention	A formulation of the conditions that must be met by an action to be satisfactorily performed. Intentions can have different extent: Motor-intentions coordinate the simplest motor actions; Proximal-intentions are at the basis of actions directed towards objects, agents, or states in the surrounding environment; Distal-intentions concern actions towards objects, agents, or states in possible (real, virtual, or imagined) worlds.
Interoception	Signals originating in the body and perceived through visceral and autonomic receptors of the nervous system.
Mediation	The use of a physical or symbolic tool (including language) to perform an action: directly on an object (first-order mediation) or to control one or more distal tools to perform an action on an object (second-order mediation; e.g. using a gamepad to control an avatar).
Narrative	Normally conceived as a sequence of events linked by temporal relations. In this paper we conceive it is a mode of cognition in which temporality is a *dominant factor* in the processing and organization of perception.
Narrative absorption	A subjective-phenomenal state that can emerge during narrative experiences and which is characterized by heightened sense of focused attention, transportation into the fictional world, emotional engagement with characters, and activated mental imagery. Similar concepts are “narrative transportation” and “narrative engagement”.
Precision	A measure of the reliability of predictions and prediction errors, which is used to control the relative influence of bottom-up prediction errors and top-down predictions, and the consequent updating of beliefs. Prediction errors with high precision have a greater impact on the reconfiguration of conditional expectations.
Predictive processing (PP)	A cognitive model that conceives perception as (Bayesian) probabilistic inference on the causes of incoming signals.
Presence	A subjective-phenomenal state usually defined as the “perceptual illusion of non-mediation” or expressed metaphorically as the sense of “being there” in some place. Here we conceive it as the state of perceived successful agency of an embodied mind able to correctly enact its own predictions.
Social presence	A subjective-phenomenal state emerging from the correct enaction of predictions regarding the actions and intentions of other agents. This process reinforces the agent’s self-perception as capable of existing in relation to a continuously changing environment populated, and acted upon, by other agents.

## 2. Methods

Before introducing our new model, we contextualize the topic under discussion within the various disciplines considered and outline areas of common interest ( section 3). Then, we introduce the results of a categorization of the items of the questionnaires most used in empirical research about presence and related concepts ( section 4). We use these results as the basis to create a new theoretical model compatible with widely used empirical instruments. We look at the similarities among self-reported measures used to assess subjective experiences with books, VR, and other media (cf. Schlochtermeier *et al.*, 2015). A general overlap of items, already acknowledged by Schubert & Crusious (2002), is a signal that similar kinds of experiences (or the same aspects of an experience) are studied in different fields, even though different names are used. That is, if the same questions are asked when participants use VR or read a short story, then either we are investigating a similar psychological phenomenon, or our instruments are badly calibrated.

Pianzola *et al.* (2021a) did a scoping review of the questionnaires used to measure presence, narrative absorption, immersion, and flow, highlighting similarities and overlaps in the wordings of items. Twenty-three questionnaires have been selected as the most used in empirical research since the year 2000, after screening 47 scales identified through three sources: the aggregator Google Scholar, the bibliography of the International Society for the Empirical Study of Literature (IGEL), and the measurement guides provided by the International Society for Presence Research (ISPR). Looking at the overlap of questionnaire items is a good starting point to identify which aspects are intersubjectively acknowledged as central to psychological phenomena like spatial presence, social presence, and narrative absorption. Similarities between concepts are usually explored looking at theoretical constructs (cf. van Baren & IJsselsteijn, 2004; Busselle & Bilandzic, 2017; Paiva de Oliveira *et al.*, 2016; Reddy, 2016; Skarbez *et al.*, 2017), but analyzing questionnaire items has the advantage of showing how the identification of psychological phenomena is operationalized, regardless of their different naming across various disciplines.

In section 5, we present a new integrated model of presence, social presence, narrative absorption, and flow, using the categorization of items introduced in section 4. We then focus on a few mediated experiences with VR and written narrative to exemplify how predictive processing can effectively explain the cognitive and affective processes related to presence, narrative absorption, and flow. We also discuss occasions in which such states are inhibited or interrupted, and eventually provide suggestions about how to empirically test our new model ( section 6).

## 3. Theory

### 3.1 VR research and narrative research

The spread of presence-related research started in the 90s, when the term *sensorimotor immersion* was introduced in telerobotics engineering to describe the kind of experience humans have when operating machines remotely, through technological interfaces, or when interacting with computer-generated virtual environments. Sensorimotor immersion is characterized by three features: the extent of sensory data, the control of sensors, and the ability to modify the environment ( Sheridan, 1992). For instance, VR is often considered an immersive medium inasmuch as it offers audiovisual data spatially organized all around the user, who can often use controllers to interact with objects in the VR space. Thus, in this context, *immersion* is a term used to refer to the technical qualities of the medium. Consequently, later VR research on presence – which is a concept referred to a subjective experience, not a property of the medium – has frequently over-emphasized the role of technological variables. A widespread belief – attested in the self-reported measures used to assess presence (e.g. Lessiter *et al.*, 2001; Lombard *et al.*, 2000; Witmer & Singer, 1998) – is that the more the interaction with a VR medium is perceived as realistic and natural, the higher the sense of presence is.

Another influential model of presence related to this conception is the one introduced by Mel ( Slater, 2009; Slater, 2018). In this model, presence in virtual reality is “the extent to which people respond realistically within a virtual environment, where response is taken at every level from low-level physiological to high-level emotional and behavioural responses” (2009, p. 3555). This perspective, also known with the acronym RAIR (response-as-if-real) is the result of two different illusions: Place illusion (PI), the qualia of having a sensation of being in a real place, and Plausibility illusion (Psi), the illusion that the scenario being depicted is actually occurring. A corollary of the RAIR vision is that is impossible to experience more presence in doing the same thing in virtual reality than in reality. However, as Villani & colleagues (2012) showed, a virtual experience could elicit a higher sense of presence if its meaning and emotional engagement are higher than in a real-life experience. In other words, presence is a multi-faceted construct also involving personal expectations and meaning attribution ( Riva & Mantovani, 2000). This is also true for reading fiction, when readers can perceive a strong sense of presence in a story world that they imagine ( Biocca, 2003; Schubert & Crusious, 2002).

To accommodate the variety of experiences in which we can feel presence, a three-poles model has been proposed in contrast to the dichotomic view of real world vs. virtual worlds. Beside real space and virtual space, the third pole of presence is that of *mental space* – emerging while reading, dreaming, or hallucinating – in which we can feel present like in a real or virtual world. The introduction of the concept of mental space is a step forward towards a more comprehensive account of presence – although we think it is not necessary (see section 3.2 below) – and it is also an acknowledgement of the existence of a research tradition that investigates phenomena similar to presence occurring with narrative artefacts like novels and films ( Gerrig, 1993; Green *et al.*, 2002). Indeed, the concept of presence has sometimes become explicitly part of attempts to describe the phenomenology of narrative experiences ( Kukkonen, 2014; Kuzmičová, 2012), or included as one of the dimensions of the broader concept of *narrative engagement* ( Busselle & Bilandzic, 2009). In other cases, similar concepts like *transportation* ( Green & Brock, 2000; Kuijpers *et al.*, 2014) and *real-world dissociation* ( Jennett *et al.*, 2008) have been used.

A second strand of VR research suggests that presence is a broad psychological phenomenon related to the control of the individual and their ^1^ social activity ( Baños *et al.*, 2000; Lee, 2004; Mantovani & Riva, 1999; Riva *et al.*, 2011; Riva *et al.*, 2015; Waterworth & Riva, 2014). More specifically, subjects are *present* when they are able to intuitively enact their embodied predictions ( Biocca, 1997; Riva *et al.*, 2015). In this sense, presence is a basic cognitive and affective process, but it is also conceived as a scalar concept, occurring with various degrees of intensity. In a similar fashion, accounts of human interaction with robots and virtual agents shifted from a focus on realistic visual appearance to models based on consciousness attribution ( Stein & Ohler, 2017). Recent cognitive narrative theories, too, explain our engagement with narrative in terms of enactment and consciousness attribution to characters ( Caracciolo, 2014; Kukkonen & Caracciolo, 2014; Kukkonen, 2019b). This broadening of theoretical scope pursues a stronger explanatory power by acknowledging the general evolutionary role of presence (cf. section 5.1), but presence remains a concept particularly useful for research on cultural and technological mediation, like with narrative and VR.

Note that the sense of the term “presence” that we described is different from “perceptual presence”, as conceived in philosophy ( Merleau-Ponty, 2012; Noë & O’Regan, 2000), i.e. the perception that objects exist as multidimensional and external to the mind, not just as a “perspectival take” on an external scene (e.g. a picture). In research on mediated experiences, presence is a subjective-phenomenal state, whereas in philosophy it is objects which are present to our senses, i.e. they are vividly perceivable. There is sometimes a theoretical confusion between the two senses, also because of influential scholars like Biocca (2001), who used the term “perceptual presence” for “telepresence”, and ( Noë, 2004, pp. 134–135), who used “virtual presence” for “perceptual presence”, in the sense of mental access to non-visible part of an object. For the purposes of this article, we consider “presence” as a subjective phenomenal state that can emerge with or without mediation.

### 3.2 The “book problem” and the “paradox of fiction”

A critical issue to consider for mediated experience is whether there is a difference between presence (often called “immersion”, in these contexts) in relation to a world displayed in VR, where users can often interact with it, from presence in relation to narrative, which concerns the way a story is told and the imagined world it elicits. In other words, shall we distinguish immersion in a story world ( Ryan, 2015) from immersion in a VR world ( Witmer & Singer, 1998)? The intuitive fact that people can perceive a strong sense of presence in an imagined world created on the basis of a very simple sensory stimulus like words on paper is known in VR research as the “book problem” ( Biocca, 2003). Empirical research highlighted some interesting differences related to this issue.

Baños & colleagues (2005) found that subjects who were asked to imagine a park felt initially more present in it, compared to subjects to whom a park was shown in a CAVE virtual environment, even though very little spatial cues were given in the imagery condition, which was mostly focused on mood induction (happiness vs. sadness). However, the sense of presence increased over time in the VR condition and decreased in the imagery condition. In a different study Gorini & colleagues (2011) tested whether VR and/or a meaningful narrative context could influence users’ sense of presence. Their results show that both VR and narrative contribute, in different ways, to elicit a sense of presence: VR increases place illusion, while narrative contributes to generating an emotional response and strengthening the subjects’ ongoing sense of presence. These results suggest that both VR and narrative are effective in eliciting a sense of presence, but for narrative it seems important that attention is not interrupted, in order to avoid breaks in presence. For instance, when the same story is read without distractions in print or in VR, the combination of written text and a 360 degrees picture (VR condition) can induce a higher sense of spatial presence ( Pianzola *et al.*, 2019).

Another experiment comparing the sense of spatial presence elicited by different writing styles ( Gysbers *et al.*, 2004) found that the baseline version of a text (e.g. “The entrance hall with a dark, wooden floor, decorated with a dark red carpet, holds a warm atmosphere, although there are only a few objects in it”) was associated with higher levels of presence compared to manipulated versions of the same text, to which more spatial cues (e.g. “It is 30 meters long, 15 meters wide and five meters high”) or instructions to imagine the space (e.g. “Try to imagine this floor as precisely as possible”) were added. To us, the basic text stimulus seems to invite a narrative experience more than the two other versions, which may be perceived as part of a memory or imagination task. This kind of text manipulation suggests once again that uninterrupted narrative progression can increase spatial presence.

To solve the book problem, Biocca suggests that “books achieve their levels of presence by making heavy use of the imagery space to ‘fill in’ the spatial model cued by the book. The details of the egocentric spatial model generated by the book are generated largely from memory. So in some ways, the presence of books contains components of the virtual space and imagery space, but unlike an immersive 3D virtual environment, there is a higher component of imagery space” (2003, p. 9). The blending of various spatial models could be a viable solution, but it is a bit of a stretch to consider the spatial model cued by the book as similar to an actual 3D virtual space. *Visual* mental imagery is not the only kind of mental imagery deployed in reading ( Esrock, 1994), and narratology often points out that fictional worlds do not give full spatial representations of everything within them ( Ryan, 2014; Stanzel, 1984). It is not necessary to postulate a mental imagery space, since all cognitive spatial models are embodied mental simulations, thus partly generated recurring to previous experiences like memories and sensorimotor scripts ( Clark, 2008). Accordingly, in contrast to Biocca, Turner (2014) suggests solving the book problem by adopting one of three possible explanations: simulation theory ( Jeannerod, 2001), emulation theory ( Grush, 2004), or mirror neurons theory ( Rizzolatti & Sinigaglia, 2008). Indeed, discoveries about brain activation during embodied simulation processes and the predictive processing model of the mind weaken the distinction between mediated experiences based on audiovisual-interactive stimuli and those based only on a verbal stimulus. We do not actually move our body while reading about the fictional character Don Quixote riding a horse, but our brain activation of sensorimotor areas is not that different than if we were actually riding a horse ( Speer *et al.*, 2009; Wilson-Mendenhall *et al.*, 2019).

The “book problem” is similar to what has been called the “paradox of fiction”, that is the fact that we respond emotionally to stimuli that we know are fictional and, therefore, are not supposed to affect us personally ( Sperduti *et al.*, 2017b; Walton, 1978). Makowski & colleagues (2019) present preliminary evidence that our emotional response system works in the same way regardless of the nature of the stimulus, but that emotion regulation “is at stake whenever engaging into fictional experiences, such as movies, books, video games, virtual environments, and possibly extending to memories and thoughts, to help us manage our emotional reaction (“it’s just a movie, it’s not for real”; “this video depicting a dramatic car crash must be a fake”)” ( Makowski *et al.*, 2019, p. 878). Emotion regulation in fictional, as opposed to real-world, contexts is an important area of inquiry closely related to presence.

Another issue related to the book problem is the “perceptual presence problem” ( Noë & O’Regan, 2002), that is our perception of objects as multidimensional despite the fact that we can only perceive a limited portion of them at a time. This “problem” is relevant for virtual/fictional/imagined objects, as well: how can we perceive them as vividly “existing” when we know they are not real? An interesting solution has been proposed by Seth (2014) through the Predictive Processing SensoryMotor Contingencies model (PPSMC), which includes counterfactual probability, unlike standard predictive processing (e.g. Rao & Ballard, 1999) and active inference models ( Friston *et al*., 2011). According to ( Seth, 2014; Seth, 2015), a vivid perception of objects is related to *counterfactual richness*, i.e. predictions about potential (but not necessarily executed) sensorimotor relations ^2^. In other words, we continuously make predictions about fictive (imagined) inputs, even when interacting with real objects. The more counterfactual predictions we make, the more our perception feels vivid. In this light, fictive stimuli are less “real” because they elicit *counterfactually-poor* predictions ( Seth, 2014; Seth, 2015). The gap is due to the lack of abundant data that could elicit predictions, because fictional, imagined, hallucinatory, and virtual worlds are structurally indeterminate, inasmuch as the provide only a limited amount of sensory data. For instance, in a novel there is a fixed number of words describing the appearance of the protagonist; and the way we can interact with an object inside a video game is determined by the amount of megabytes encoding its game mechanics. The limitation of the PPSMC account is that it downplays the role of agency (e.g. active inference or imaginative skills) and of contextual information not directly related to the stimulus, like the familiarity with genre conventions for certain books, or the personal relevance of certain inputs (see section 5.6; cf. Parola *et al.*, 2016). Conceptualising modes of agency in the mediated, fictional contexts of VR and reading literature, as they relate to presence, will contribute to a better understanding of the “perceptual presence problem”.

To sum up, in the framework of PP, the way interactive and non-interactive mediated experiences affect our spatiotemporal perception may not require distinct concepts, despite different theorizations. Regardless of the context or use of media, presence and narrative absorption are phenomena based on embodied predictive processing. In our view, the book problem can simply be solved in terms of a shift in the precision of embodied predictions towards *interoception*, rather than *exteroception*, i.e. towards the inner bodily states elicited by reading rather than the perception of external stimuli ( Kukkonen, 2019a; see Quadt *et al.*, 2018 for the distinction between interoception and exteroception in a PP framework; and see section 5.2 here). Evidence of the closeness of the various experiences of presence in different contexts is the similar wording used in questionnaires.

## 4. Measures and conceptual relations

Both presence and narrative absorption are multidimensional constructs – depending on the definitions, presence can subsume “spatial presence,” “social presence,” “realism/ecological validity,” “engagement,” or other variations of these concepts ( Skarbez *et al.*, 2017), and the questionnaires created to grasp these two states use very similar items. For instance, the narrative absorption item “When I was finished with reading the story it felt like I had taken a trip to the world of the story” ( Kuijpers *et al.*, 2014) strongly resembles the spatial presence item “After my experience of the displayed environment, I had a sense that I had returned from a journey” ( Lessiter *et al.*, 2001). The Sense of Presence Inventory (ITC-SOPI) by Lessiter and colleagues is a scale developed for cross-media use, hence it includes items covering both interaction and emotional involvement related to the perception of time. However, similarities and overlaps are quite frequent even between questionnaires with narrower scope. Table 2 summarizes these overlaps between items, our categorization, and the psychological phenomenon that we associate to it. A complete list of the grouped items (n = 308) and a more detailed discussion of the similarities can be found in ( Pianzola *et al.*, 2021a; Pianzola, 2021b).

**Table 2.** Categorization of items (n = 308) from presence, flow, game, and narrative questionnaires (reproduced with permission from Pianzola *et al.*, 2021a).

Total items	Scales with item	Item type	Category	Main psychological phenomenon
23	11	Attention (no external thoughts)	Attention	Attention
17	9	Attention (no external perceptions)		
18	11	Time distortion	Time	–
17	9	“Being there” (feelings and perceptions, not thoughts)	Space	Spatial presence
8	5	Realities overlapping		
6	3	Closeness of story world		
7	6	Return to reality		
5	5	Being part of the action (also partly overlaps with “being there”)		
10	5	Possibility of action in space	Agency	
6	4	Control of content		
5	3	Control of medium		
9	6	Naturalness/fluency of medium use		
14	6	Perceived realism	Comparison	
5	2	Attention to another agent	Attention	Social presence
5	4	Co-location with another agent	Space	
24	4	Mind reading	Cognition	
5	2	Behavioural response to another agent	Agency	
13	7	Matching of another agent’s emotions	Emotion	
4	3	Feelings for another agent		
6	5	Connection with another agent	Emotion/Cognition	
16	6	Understanding of another agent (perspective taking, cognitive empathy)	Cognition	
12	7	Challenge	Cognition	Flow
8	4	Vividness of imagery	Comparison	Narrative absorption
14	7	Comprehension of content	Comprehension	
9	6	Suspense/anticipation	Emotion/Cognition	
18	10	Emotional response to medium/content	Emotion	Emotional impact
14	7	Explicit use of involvement/engagement terms	Metaphor	–
10	9	Explicit use of absorption/immersion terms		

Items inquiring about attention and the perception of time are the most frequent ones, together with items about the emotional impact of the mediated experience. “Space”, “agency”, and “realism” are the categories most often associated to a sense of spatial presence. Many theorizations of presence consider visual realism and naturalness of interaction as core aspects, but broader psychological conceptions (cf. section 3.1 and section 5.1) and a large-scale collaborative project ( Hartmann *et al.*, 2016; Vorderer *et al.*, 2004) have excluded realism from the subdimensions of presence, keeping only “self-location” and “possible action” as core dimensions. Inquiring about the realism of computer graphics – but also about the vividness of the imagery elicited by a book – is a way to check how similar the mediated/imagined experience is to a non-mediated one. These comparisons do not seem to help to explain the underlying psychological processes that bring to the emergence of a sense of presence. For example, they cannot explain how a virtual experience could elicit a higher sense of presence than a real one ( Villani *et al.*, 2012).

The label “social presence” can describe the second macro group of items identified. The basic principle at stake is that perceiving the existence of other agents can affect our sense of spatial presence. Another aspect is that our perception and experience can be intensified when interacting with (real or fictive) others or following their actions. Noticing the existence of others, interacting with them, and responding to and understanding others’ mental states are all aspects informing social presence. Items associated to social presence often co-occur with spatial presence items and seem to entail spatial presence as the basis on top of which social presence can emerge. These conceptualizations interpret social presence as a form of spatial presence in co-participation. Indeed, spatial presence seems to be part of all the other phenomena considered, since its items often appear in questionnaires about flow experiences, narrative, and games. In the case of flow, consistently with the original theorization by Csikszentmihalyi (1990), there is always the addition of a specific group of questions inquiring about the sense of challenge offered by an experience. Lastly, narrative absorption is characterized by spatial presence and social presence with the characters of a story, with the addition of imagery, the feeling of suspense triggered by narrated events, and the comprehension of the content of a story. The latter aspect is sometimes articulated in terms similar to that used for flow, namely the challenge offered to the audience.

**Figure 1.**
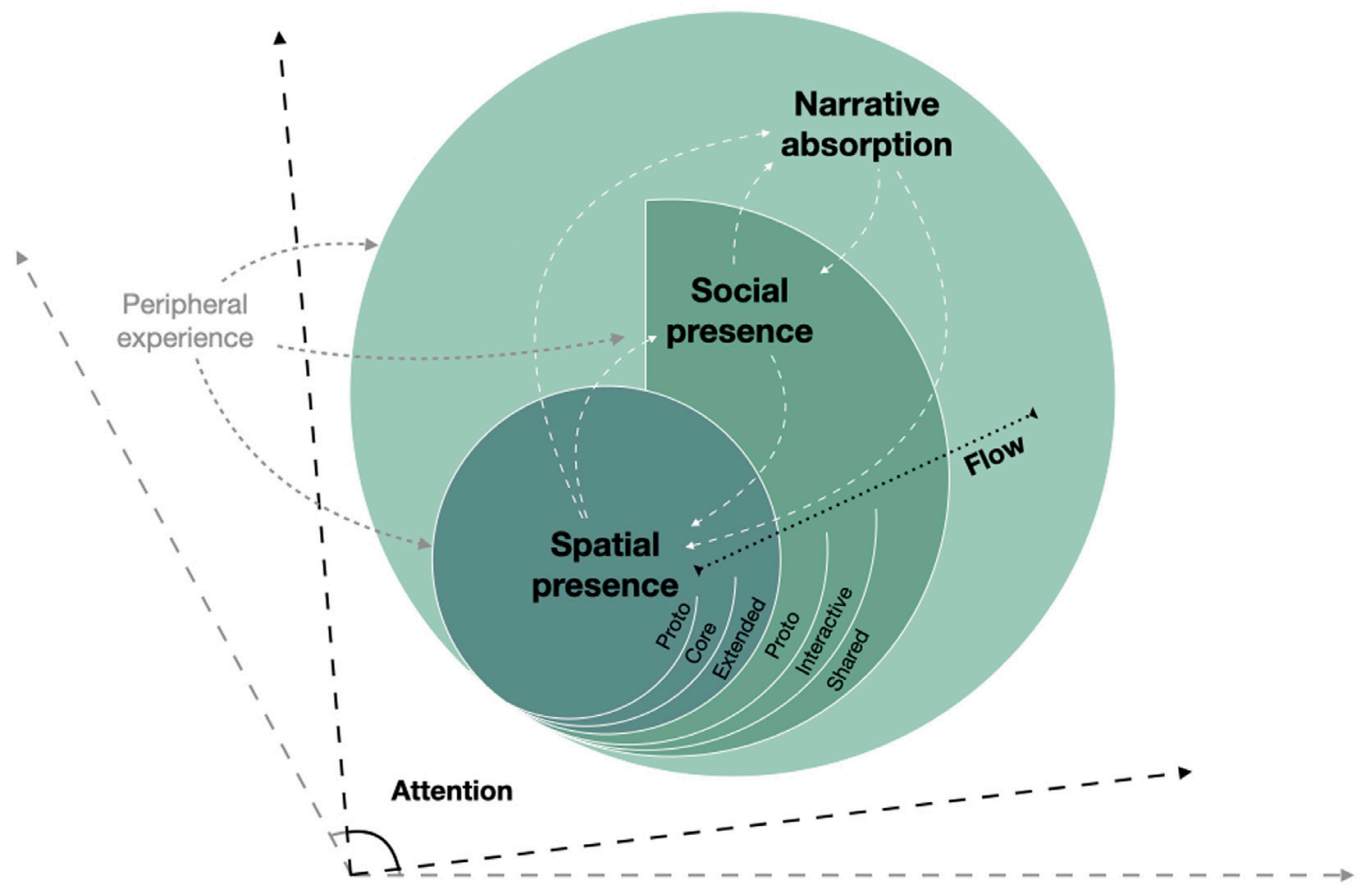
Visual representation of the hierarchical relations between spatial presence, social presence, narrative absorption, and flow. Phenomena of wider scope include phenomena of narrower scope as subdimensions, but social presence is not strictly necessary for the emergence of narrative absorption, although it is often involved. White dashed lines represent feedback loops between phenomena of different scope, meaning that phenomena of higher order can intensify phenomena of lower order: e.g. elements contributing to the emergence of narrative absorption (like, suspense) can intensify the sense of spatial presence. The subtypes of presence and social presence (proto-, core-, extended-) are also represented, ordered by the extent of their scope (cf. section 5.1 and Section 5.5). Focused attention is the precondition for the emergence of all the represented phenomena. Peripheral experience is outside the scope of selective attention, but it can nevertheless intervene in the emergence of presence-related phenomena, for instance when the crowd is cheering to support an athlete’s performance (cf. section 5.4).

The review presented suggests a possible cross-disciplinary systematization of the various concepts ( Figure 1). Attention and an altered perception of time are common to all the considered phenomena. Spatial presence is the phenomenon at the core of all mediated experiences and concerns spatial location and agency. Social presence and narrative absorption are phenomena of increasingly broader scope, each of them including the phenomena of narrower scope. Flow is a concept specifically related to the balance between a person’s skills and the complexity of a perceptual stimulus, thus it can characterize all kinds of experiences. In the next section we present a model compatible with this categorization, integrating previous research on the psychology of VR and communication – by Riva and Mantovani ( Riva, 2018; Riva *et al.*, 2015; Riva & Mantovani, 2014) – and on narrative – by Kukkonen (2020) and Pianzola (2018).

## 5. New model

### 5.1 Spatial presence

According to PP the brain continuously updates a model of the relations between the body and the space around it, generating predictions about the expected sensory input (probability) and trying to minimize the amount of prediction errors. More specifically, our brain functioning can be explained with a hierarchical generative model that performs a Bayesian form of inference elicited by the available sensory data, generating sensorimotor hypotheses (predictions) about the most likely causes of the data.

Predictions are mainly simulations of bodily states and they include visceral/autonomic (interoceptive), motor (proprioceptive and vestibular), and sensory (e.g., visual, auditory) information ( Riva, 2018). Moreover, embodied simulations reactivate multimodal neural networks, which have produced the simulated/expected effect in previous occasions. PP works not only for actions, but also for language and affect (basic emotions, feelings, moods, etc.). When we use language, a group of distributed multimodal patterns of activity across different populations of neurons (motor, somatosensory, limbic, and frontal areas) that support achievement of a goal are activated. Accordingly, the simulation of a concept or a situation described through language involves their re-enactment in modality-specific brain areas. In a similar way, the brain also uses emotion concepts to categorize sensations by correctly anticipating (predicting and adjusting to) incoming sensations and using past experiences of an emotion to categorize the predicted sensory array and guide action ( Barrett, 2017; Seth, 2013; Van de Cruys, 2017). As suggested by Seth & Friston (2016): “emotional content is determined by beliefs (i.e. posterior expectations) about the causes of interoceptive signals across multiple hierarchical levels” (p. 5).

One of the first accounts of presence in terms of PP has been proposed by Seth *et al.* (2012), for whom “presence is the result of successful suppression by top-down predictions of informative interoceptive signals evoked (directly) by autonomic control signals and (indirectly) by bodily responses to afferent sensory signals” (p. 2). A tightly related and complementary state is *agency*, which emerges from successfully embodied predictions about exteroceptive and interoceptive signals mediated by the sense of presence. Agency is functionally localized at a higher hierarchical level than presence, so that prediction errors related to presence influence the sense of agency, while predictions and prediction errors concerning agency do not intervene in the perception of a sense of presence. Seth and colleagues reverse the relation acknowledged by the majority of research, according to which agency is a subdimension of presence, thus located at a lower hierarchical level (see Table 2 above). The problem is that, in this way, agency can constrain predictions about presence, because “low-level perceptual content is determined via a cascade of predictions flowing from very general abstract expectations which constrain successively more detailed (fine-grained) predictions” (p. 6). But we will show that predictions related to presence can be successfully enacted even in the case of prediction errors related to agency (see section 5.4). In our model, presence is hierarchically higher and can constrain the perception of agency, namely an agent can feel present even though they fail in enacting some motor intentions.

**Figure 2.**
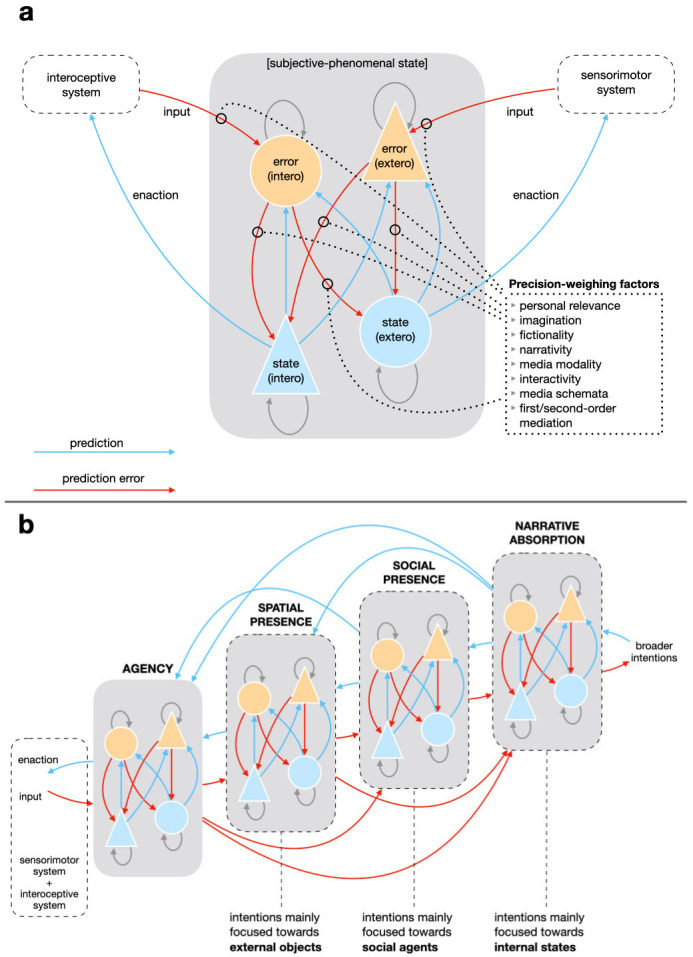
Predictive processing model of spatial presence, social presence, narrative absorption, and their relation with agency. Panel ( **a**) presents the dynamics of prediction and prediction error in relation to exteroception and interoception. This model is valid for all the subjective-phenomenal states presented in panel ( **b**). Each component comprises state (light blue) and error (orange) units for both exteroception and interoception. State units generate control signals (enaction) and predictions (light blue arrows) about the consequent input signals; error units compare predictions with afferents, generating error signals (red arrows). The precision of error signals is weighed by different factors related mainly to mediation and the medium format, but also to personal differences ( Kidd & Castano, 2017; Samana *et al.*, 2009). Panel ( **b**) shows the hierarchical relations between the various subjective-phenomenal states. Hierarchically higher states can constrain predictions generated by lower-level states, intensifying or inhibiting them. Intentions are crucial for distinguishing between the different presence-related phenomena: it is mainly the processing of intentions oriented towards external objects in comparison to exteroceptive signals that enable spatial presence; intentions oriented towards other agents and updated on the basis of their actions enable social presence; and intentions oriented towards interoceptive states enable narrative absorption.

We conceive presence as a neuropsychological phenomenon whose goal is to *generate* in the embodied self a sense of agency ( Riva *et al.*, 2015) that supervises predictions related to both exteroceptive and interoceptive perception ( Figure 2). An agent feels present when they are able to correctly and intuitively enact their embodied predictions. Thus, presence is a general embodied cognitive state, not entailing any specific object or location ( Seth *et al.*, 2012). It is rather an expression of the self-perceived skilful agency of an embodied mind, and it is hierarchically higher than agency.

In this view, the sense of presence can be considered as an evolutive tool used to track the difference between predicted sensations and those coming from bodily stimuli, both external and internal ( Riva *et al.*, 2015). The extent of this difference corresponds to the degree of self-perception as a skilful agent: presence. In order to successfully achieve the goal set by their intentions (both pre-reflexive and conscious), an agent tries to correctly predict their own behaviour and overcome any breakdown in their activity. Accordingly, this leads the agent to often look for situations which are likely to increase the chance of successful action ( Csikszentmihalyi, 1990). The rationale behind this evolved capacity is the achievement of effective free-energy minimization ( Friston & Stephan, 2007). And the rate of error minimization over time, if steady and connected to relevant intentions, may induce “a sense of properly functioning bodily and sensorimotor systems”, which can be experienced as emotional states ( Riva *et al.*, 2007; Van De Cruys, 2017; cf. Makowski *et al.*, 2017) and is usually described as “flow” ( Csikszentmihalyi, 1990).

The process of minimization of the level of free-energy through the correct prediction of incoming exteroceptive/interoceptive inputs has two clear evolutive goals ( Riva & Waterworth, 2014; Waterworth & Riva, 2014): on the one hand, the self uses it to overcome any threat or breakdown in its activity (break in presence); on the other hand, the self looks for activities that maximize the agent’s environmental and social fitness. Bruineberg & Rietveld’s (2014) notion of “optimal grip” indicates a conceptual link between free-energy minimization and flow, as an optimal experience that emerges when mastering the challenges of an environment. By cultivating optimal experiences, the subject is able to express, enact and recognize increasingly more complex intentions. This is achieved through the successful use of new tools and the successful exploration of new spaces, including simulated spaces (virtual, imagined, and fictional). The enactment of predictions is not limited to non-mediated bodily experiences, it also occurs through physical or digital tools, as well as through symbolic and cultural ones ( Riva & Mantovani, 2014). For instance, as suggested by ( Slater, 2009; Slater, 2018), a VR world mimicking the “real world” will be designed to let an agent predict the sensory consequences of its movements, providing in response the same scenes and effects they would see in the “real world” (see section 5.3). However, understanding presence also requires understanding the link between agency, intentions, and the tools used to enact them.

### 5.2 Agency, intentions, and presence

The American philosopher John Searle, in his cognitive approach to agency, defined action as the sum of two components, intention and movement ( Searle, 1983). Intention “describes” the conditions that must be met by the action to be satisfied. Movement concerns the means by which the success of the intention is verified. There is a direct link between Searle’s definition and the concept of presence: intentions can be defined as predictions of action-effects that activate and guide our movement. A similar assumption underlies the enactive strand within 4E cognition, where it is argued that we perceive the world through its sensorimotor contingencies, that is, the way in which we could interact with it ( Noë, 2004). Enactivism does not foreground intention, claiming that most of these processes are preconscious. However, the potentiality of interaction can be understood as the potentiality of action-effects, and this holds close connections to embodied predictive processing ( Seth, 2014).

As Searle noted, not all intentions are the same. When I move my body (intention-in-action), the action itself is the condition of satisfaction, i.e. my intention to move my hand is satisfied if I can move my hand. When intentions are oriented towards external objects, they are satisfied by the overlap between the intentional disposition (prior intention) and the result of the action, i.e. my intention to take an apple is satisfied if I move my hand, and the hand grasps the apple. In addition, as emphasized by Bratman (1987) and Pacherie ( Mylopoulos & Pacherie, 2019; Pacherie, 2006; Pacherie, 2008), prior intentions can be further distinguished in present-directed ( *proximal*) and future-directed ( *distal*) intentions according to the moment in which the intention will be satisfied, i.e. now or in the future.

Another difference in prior intentions was suggested by The Centre for Cognitive Sciences in Turin, Italy ( Bara *et al.*, 2011; Ciaramidaro *et al.*, 2007), namely between “private intentions” and “social intentions”. Private intentions are all of the prior intentions that can be satisfied by the individual alone. Social intentions are all of the prior intentions that involve at least one other person whose active participation is necessary for the intention to be satisfied. Relatedly, Searle introduced a specific kind of social intentions, i.e. “collective intentions” (We-intentions), which call for a form of cooperation that is not a mere coordination between subjects; rather, it involves mutual understanding of other participants’ intentions ( Searle, 1990). Namely, collective intentions include one or more prior intentions that describe the subject’s personal contribution to the collective action, i.e. “I intend to do action X as part of the group’s action Y”.

This brief analysis of the characteristics of intentions suggests a possible structure of human intentionality that includes seven types of intentions organized on three levels:

- Motor Intentions coordinate the simplest motor actions (not directed towards an object), such as opening the hand or protruding the lips.
- Private, Social and Collaborative *Proximal* intentions are at the basis of actions directed towards states, objects, or agents in the surrounding environment. They may be private (“pick up the apple”), social (“take my sister’s hand”), or collaborative (“assemble an Ikea furniture”). These intentions are the result of the interaction between subjective needs and the surrounding physical and social environment.
- Private, Social and Collaborative *Distal* intentions are at the basis of our actions towards states, objects, and agents in possible worlds (non-actual worlds linked to the actual one by a relation of accessibility). These intentions may be private (“lose weight”), social (“going to watch a play at a theatre”), or collective (“start a family”). These intentions are the result of the interaction between subjective needs and the possible worlds opened to the subject by the culture of reference.

In this view, any intentional level has its own role, i.e. motor ( *Motor intentions*), situational ( *Proximal intentions*), and volitional ( *Distal intentions*) guidance and control of action. In addition, they form an intentional cascade ( Pacherie, 2006; Pacherie, 2008) in which higher intentions generate lower intentions.

The link between intentions, predictions, and actions with respect to spatial presence can be better understood if we consider that the evolution of the sense of presence is phylogenetically and ontogenically related to the one of the self ( Damasio, 1999; Riva *et al.*, 2004; Riva *et al.*, 2015; Triberti & Riva, 2016; Waterworth & Riva, 2014). The first type of spatial presence that emerged is *proto presence*, a process of internal/external separation, which is related to basic motor intentions ( *M-intentions*) and perception-action coupling (self vs. non-self) ( Table 3). That is, the more an agent is able to correctly couple perception and action, the more they differentiate themselves from external stimuli, increasing their ability of body orientation in space. Questionnaires’ items inquiring about location in space investigate proto presence (cf. Table 2). The second type of spatial presence that emerged is *core presence*, an agent’s intentional orientation of sensory selective attention towards perceptions (self vs. present perceptions). That is, the more an agent is able to focus on their bodily experience, the more they are able to perceive their own agency and their own current goal-directed proximal intentions ( *P-intentions*). The questionnaires’ items inquiring about someone’s agency usually investigate core presence, namely those items about the possibility of action. The third type of spatial presence that emerged is *extended presence*, an agent’s evaluation of the significance of the current experience (self in relation to the present situation). That is, the more an experience is coherent with an agent’s future goals ( *D-intentions*), the more the agent is feeling present in it. Some of the questionnaires’ items inquiring about the sense of agency investigate extended presence, namely those items about the control of medium/content and the fluency of media use, because these aspects involve the ability to predict possible actions. Often, this presupposes a familiarity with the medium/content ( IJsselsteijn, 2003) and its relevance for the agent’s current or future life.

**Table 3.** The types of spatial presence and their relations to the subject and to mediation (reproduced and modified with permission from Riva *et al.*, 2004).

Type	Relation to self	Consciousness	Intentions	Mediation (form/content)
Proto presence	Self vs. non-self (other)	Mostly unconscious	Motor intentions	Proprioceptive (spatial presence/ enaction)
Core presence	Self vs. present perceptions	Conscious here and now	Proximal (present) intentions	Perceptual (sensory presence/enaction)
Extended presence	Self in relation to the present situation	Conscious of self in relation to situation	Distal (future) intentions	Conceptual (intellectually/emotionally significant content)

Similarly, the challenging aspect characteristic of flow experiences is strictly related to extended presence, since it depends both on: (i) the ratio between challenge and skills at stake in the experience – i.e. the ability to predict possible actions and outcomes that minimize the amount of free-energy – and (ii) the goals of an agent. For instance, (i) if a game is too difficult or a book is in a foreign language that I do not know well, extended presence and flow are limited; if a game is too easy or a book is too simple, I do not feel challenged. If (ii) the genre of a videogame/book is not coherent with my goals – e.g. it is a shooter game, not a desired platform game; it is a fantasy book, not a desired crime novel – I perceive the experience as not relevant for my intentions and goals (prediction error).

Mylopoulos & Pacherie (2017) suggested that the link between intentions and the different embodied predictions is achieved through motor schemas. Namely, intentions include executable action concepts that describe the organization and structure of the action (motor schema) using a set of predefined parameters related to the body and the surrounding environment ( Jeannerod, 1997); these parameters can be defined and updated using Bayesian inference and modelling ( Braun *et al.*, 2010). However, according to Shepherd (2019) intentions have a dual format: “Intentions can take propositionally formatted contents that enable their integration with propositional thought. And intentions have motorically formatted contents that communicate in a fairly direct way with the operations of motoric-level action implementation” (pp. 294-95). Empirical evidence that supports the existence of a link between motor schemas and propositionally formatted contents is the activation of motor representations during the processing of linguistic items pertaining to action ( Buccino *et al.*, 2016; Kemmerer, 2015; Repetto *et al.*, 2013). Here we suggest that propositional content can be involved in the emergence of spatial presence, but it is not strictly necessary.

When an agent is learning an action (e.g. to drive a car or to speak a new language), the propositionally formatted content of the action is matched consciously with the embodied formatted contents to minimize the level of free-energy. For example the P-intention “Adjust the seat in regards to the pedals” within the D-intention “Learn to drive a car” is initially associated with an embodied representation including ( Tate, 2019; Seth & Friston, 2016): (i) a high-precision prediction of the motor consequences of moving the body (e.g. the feet and the steering wheel); (ii) a low-precision prediction based on the propositional content of the action-properties of external objects and environmental features (exteroceptive predictions) (e.g. object shape and size of the pedals and the seat, distance, possible movements); (iii) the changes in internal body states (interoceptive predictions) (e.g. heart rate, blood pressure) determined by the beliefs about a specific object (e.g. if the agent is too far from the pedals and cannot use them in the right way, they can have a car accident). When, after the training, the agent is able to intuitively drive the car, the propositional content is only used to describe the intention, not to enact it. This aspect is particularly important in relation to mediated experiences involving story worlds, since the represented world is a kind of propositional content. According to the model described above, even in the case of propositional content represented by the medium, it is not necessary to postulate that there is a propositional content generated by the mind and intervening in all predictive processing. Rather, some of the embodied predictions drive the intuitive action of the agent and are the result of a process of inductive generalization from sets of motor representations or from sets of already extant motor schemas ( Mylopoulos & Pacherie, 2017). This link also suggests a critical role of intentions in shaping peripersonal space, a concept useful to describe mediated experiences.

Rizzolatti and colleagues defined “peripersonal space” (PPS) as the space immediately around the body ( Rizzolatti *et al.*, 1996), and later studies demonstrated both its role in monitoring the position of objects in space in relation to the body, and its plasticity after both short-term and long-term learning and practice with a tool ( Holmes & Spence, 2004). Recent studies, however, have provided a more complex outline of the role of PPS. Namely, (i) the affordances of an object evoke a motor response in the observer’s brain even when it is out of their reach, provided that it is reachable by another individual ( Cardellicchio *et al.*, 2013; Fini *et al.*, 2014); (ii) the modification of the PPS after the use of a tool does not depend strictly on the active use of the tool itself, but it is triggered by anticipatory images of its action-effects ( Galli *et al.*, 2015); (iii) the plasticity of the PPS is affected both by psychological characteristics of the individual, e.g. trait anxiety ( Spaccasassi & Maravita, 2020), and by psychological properties of the objects, e.g. affective valence and knowledge about their functions ( Valdés-Conroy *et al.*, 2012).

Taken together these studies suggest that intentions, through PPS, filter the representation of the potential motor acts afforded by visible objects, enabling their identification as potential targets for one’s own actions or others’ actions ( Maranesi *et al.*, 2014; Serino, 2019). As suggested by Bufacchi & Iannetti (2018), PPS can be reconceptualized “as a set of graded fields describing behavioural relevance of actions aiming to create or avoid contact between objects and the body” (p. 1076).

### 5.3 Mediated action

The boundaries of PPS can also be extended by external tools and other individuals: “technologies, used to push our action possibilities beyond the standard limits of our physical body, plastically reshape PPS representations… At the same time, it is also possible that emotional and social factors emerging from face-to-face interaction with others in turn modulate PPS neurons’ ability to map the space around the others into one’s own PPS” ( Serino, 2019, p. 149-150). To sum up, the enactment of predictions can occur mainly in three ways ( Riva & Mantovani, 2012; Riva & Mantovani, 2014):

- through the body in a non-mediated way;
- first-order mediation: through a proximal physical or symbolic tool (an artefact directly usable by the body, including language) to exert an action upon an object;
- second-order mediation: through a proximal physical or symbolic tool (an artefact directly usable by the body, including language) that controls one or more distal ones (e.g. a tool perceivable in the extrapersonal space, such as an avatar or a narrative) to exert an action upon an external object (e.g. using a gamepad to control an avatar in a videogame) or a mental object (e.g. giving sense to a narrative or solving a thought experiment).

The way in which embodied predictions are enacted has different effects on an agent’s sense of presence. With a successfully predicted first-order mediated action the proximal tool extends the peripersonal space of the acting subject. In other words, the acquisition of a motor skill related to the use of a proximal tool extends the body model used to define the near and far space. From a neuropsychological viewpoint the tool extends the near space until the tip of the tool. From a phenomenological viewpoint, instead, the tool is now “incorporated” by the agent, who can use it intuitively as they use their hands and fingers, thus being able to feel spatially present while using it. A successfully predicted second-order mediated action produces an additional body simulation centred on the external tool. Second-order mediated actions are based on the simultaneous handling of two different body models – one centred on the body and a second one centred on the external tool – that are weighed ( *precision*) in a way that minimizes uncertainty during the mediated action. In other words, the second PPS centred on the external tool competes with the one centred on the body to orient action and perception. Namely, when the externally-centred PPS becomes the most relevant one, it shifts the body-centred space to the one surrounding the external/symbolic tool. From a phenomenological viewpoint, the agent is now present in the external space and can act in it intuitively.

The extension of the PPS boundary is also affected and regulated by precision attributed to highly-structured predictions – like the previous knowledge of what a certain medium is and affords (“media schemata”, IJsselsteijn, 2003) – and to interoceptive signals. In general, interoception supports the balance between stability and adaptability of the bodily self by increasing the stability of the modelling of near-body space, while leaving the representation of far-body space potentially adaptable to all contexts. “Less attentional resources allocated to far exteroceptive stimuli, compared to bodily interoceptive input, may play a crucial role in this balance between stability and adaptability of PPS boundary in high interoceptive individuals” ( Ardizzi & Ferri, 2018, p. 84). However, there is yet no evidence that highly interoceptive individuals are more successful in enacting second-order mediated actions ( Kukkonen, 2019a).

The precision attributed to interoceptive predictions and prediction errors is higher for symbolic tools: using language (proximal tool) is a first-order mediated action that, when successfully predicted, can enable the use of words to express and communicate inner states, extending the interoceptive accuracy of an agent. When speaking a mastered language and interacting with others using such language, an agent feels a stronger sense of agency and is able to better perceive, express, and interpret interoceptive stimuli: the agent feels present because the correct prediction of the inner states increases the stability of the modelling of the near-body space. On the other hand, reading fiction is a second-order mediated action in which an agent uses language (proximal tool) to enact a character’s (distal tool) action in the fictional world ( Caracciolo, 2014), and this enaction has an effect on the agent’s interoceptive states (object). A reader who successfully enacts a character’s perceptions is focused on their own (the reader’s) interoceptive states (“interoceptive attunement”, Kukkonen, 2019a) and feels present because of it. However, since space is a useful category for perceiving and interpreting one’s own actions and states, it is much easier to metaphorically say that the reader is present in the fictional world in which the character is acting (cf. Lakoff & Johnson, 2008). As noted by Kukkonen (2019a): “In the process of interoceptive attunement … a balance is struck between the embodied signature of the text and readers’ own body state. Such a balance would also inform the degree of empathy we feel for characters” (p. 120).

With both exteroception-focused and interoception-focused second-order mediated actions, an agent has two intentions: the first one is of the kind “to use the gamepad to move the avatar”, or “to use language to enact and give sense to the actions of the character” (proximal intentions); the second one is of the kind “to strategically control the avatar in order to succeed in the game”, or “to understand the character’s intentions” (distal intentions). The latter happens by progressively (re)constructing the *probability design* of the novel and using it to make accurate predictions of what a character will do or feel ( Kukkonen, 2020; cf. section 5.6). Through the story, a reader progressively learns to enact the character’s present intentions taking into account the future intentions envisaged in the narrative organization (plot, genre conventions, tropes, etc.). This results in an increased sense of agency, because the reader perceives themselves as able to master mediated and simulated exteroception and interoception. Moreover, it can be the case that successful predictions of the narrative’s P- and D-intentions match a reader’s own P- and D-intentions, e.g. the desire to read a suspenseful romance and the belief that everyone has a “soul mate,” like it may be narrated in the story. In this situation the sense of presence can be further intensified, since the reader successfully performs two actions (for an example, see Gorini *et al.*, 2011): interpreting and accepting (“incorporation”, “suspension of disbelief”) the narrative’s organization (presence influenced by first-order mediation) and confirming their Self-perception after an exploration of their own feelings and beliefs, prompted by the narrative (presence influenced by second-order mediation).

The dynamics of presence also involve socio-culturally distributed and embodied interactions which can be referred to as *enculturated predictive processing* ( Fabry & Kukkonen, 2019; Roepstorff *et al.*, 2010). The neuropsychological enculturated processes related to presence evolved in response to sensory stimuli but also through learning processes which are shared and transmitted within communities of agents. For instance, concepts related to predictions develop through the learning of language ( Barrett, 2017) and through cultural learning of embodied habits and practices ( Roepstorff *et al.*, 2010). Enculturated predictive processing works similarly in mediated and non-mediated experiences but there are some differences between interactive media (VR, games), audiovisual media (films), and textual media (novels). To better describe the complexity of mediated experience with these kinds of cultural artefacts, it is useful to first outline other cognitive processes involved and that are central for many measurement tools, like attention and emotions (cf. section 4).

### 5.4 The role of attention and interoception

Attention is a by-product of multiple interactive processes, involving a network of neural systems, related to the selection of information and to behavioural response control ( Cohen, 2014). An extension of the predictive processing model ( Feldman & Friston, 2010) suggests that attention is inference about the uncertainty (or precision) of the causes of sensory inputs. In particular, attention optimises the expected precision of predictions by promoting the neural encoding of prediction errors ( Smout *et al.*, 2019). In this view, attention, by defining the precision of the different layers of predictions, is a critical dimension of presence.

*Sensory selective attention* operates by selecting certain stimuli over others and orienting predictive processing towards the selected stimuli. It is a precondition for the emergence of all three types of presence (proto, core, extended), but in different ways. Sensory selective attention can operate either in a reflexive automatic way, when elicited by environmental stimuli (exogenous attention), or as part of an intentional goal-directed action (endogenous attention) ( Cohen, 2014; Posner, 2004). In the former case, only M-intentions are involved; in the latter case, P- and D-intentions are involved, too. Moreover, in order to perform tasks that are part of presence-inducing experiences, other kinds of attention are often required. That is, after sensory selection, cognitive and physical resources are allocated based on the prevailing task demands, and the intensity of attention changes accordingly ( *focused attention*). Depending on the kind of activity, these processes can extend over a relatively long period of time ( *sustained attention*). Attention, perception, prediction, and enactment of intentions enable the emergence of presence, which in turn (feedback loop) can intensify the focus of attention (cf. Cohen, 2014). This intensified focus of attention then influences perception, the predictions an agent (consciously and unconsciously) makes, and the enactment of goal-directed intentions.

When a prediction error occurs, an agent can experience a break in the sense of presence. A stimulus that is different from what was expected is surprising and attracts sensory selective attention, because a new updated prediction is needed ( Friston, 2010). For instance, consider someone playing a VR game in which they are trying to move a cup from the table and throw it on the floor in order to complete a task in the game. Suppose they feel a strong sense of presence, since the experience is fun and coherent with the agent’s D-intentions of having a good time. If the object floats mid-air instead of dropping on the floor, this can cause a break in *extended presence* because it is not coherent neither with the real-world model – and the predictions based on it (M- and P-intentions) – nor with predictions made to complete the task in the game. However, such a break intensifies and directs exogenous sensory selective attention, because the disruption of the laws of physics is a prediction error that compels the agent to gather more sensory data and make new predictions to explain away the errors in their embodied simulation. In this situation, despite the break in extended presence, it can be that proto and core presence are not disrupted, for instance, if the act of grasping and holding the cup is coherent with M- and P-intentions and the floating of the cup can have some magical or technological explanation that is coherent within the game. This would be a case of a minor break in (extended) presence, causing exogenous sensory selective attention to partly shift toward the element that caused the prediction error – the floating cup – while endogenous (voluntary) sensory selective attention remains focused on completing the task of moving the object as part of the game (D-intentions). The focus of attention on *extended presence* can relativize prediction errors (assigning lower precision to them), consequently minimizing breaks in *core presence* (for an experimental example, see Spagnolli & Gamberini, 2006).

A disruption of *core presence* can occur when the controller-object interaction does not work as expected, e.g. the agent cannot grab the cup properly. In this case, exogenous sensory selective attention can cause endogenous attention to shift outside the current range of focused attention: from the interaction with the object within VR to the controller as an inaccurate bodily extension. The focus shifts from the movement of the object as part of the VR game (D-intentions) to the use of the controller (M- and P-intentions), whose glitch the agent wants to figure out. In contrast to the previous example, this is also a major break in *extended presence* – a phenomenon of broader scope and entailing *core presence* – since the prediction errors concerning M- and P-intentions cannot be explained away as part of the game mechanics. A more extreme case of a break in *proto* and *core presence* would be bumping into a chair while wearing a VR headset, since predictive processing is based on M- and P-intentions with respect to the peripersonal space of the VR game, and the chair is not part of it.

So far, we have presented examples regarding VR, in which sensory selective attention is mainly direct towards exteroception, but there are cases in which attention is directed towards interoception, namely affective and imagined states. Interoception includes a range of perceptions related to: brain-to-body signalling; neural encoding, representation, and integration of information concerning body states; influence of basic inner perceptions on more complex states (including feelings and emotions); and conscious perception of bodily states ( Quadt *et al.*, 2018). The fun and the challenging sensation perceived by the agent in the VR game example above are related to interoception. In general, humans tend to keep interoception stable in order to minimize prediction errors when facing new situations ( Pezzulo *et al.*, 2015). The homeostasis between these two modes of embodied perception allows an agent not to continuously update predictions about their Self, leaving more cognitive resources for exteroception. This is also evident in the example above, in which interoception is steadily focused on the goal of the game, while exteroception fluctuates, trying to update predictions about external stimuli. The inverse case can also occur, typically when reading. Reading is an activity during which exteroception is stable – usually done while sitting, moving only eyes and hands – and sensory selective attention is mainly directed towards interoception ( Kukkonen, 2019a). Different media establish different points of balance between interoception and exteroception; reading stabilizes the exteroceptive side of the balance, enabling the exploration of inner bodily states elicited by the narrative, which are related to the reader’s enactment of characters’ perceptions, along with emotions and memories associated to them ( Caracciolo, 2014).

To solve the “book problem” it is not necessary to postulate an imaginary world in which an agent feels present, this is just a metaphorical expression used to describe a complex phenomenon. The sense of spatial presence experienced when reading or engaging with audiovisual narrative, can be explained in terms of a shift of sensory selective attention from exteroception towards interoception, and increased precision attributed to interoceptive predictions ( Kukkonen, 2019a), which is also relevant for discussions of mind-wandering in reading ( Fabry & Kukkonen, 2019; Smallwood & Schooler, 2015). The underlying neuropsychological dynamics are the same as for VR and non-mediated experiences, involving predictive processing and enactment of intentions. In the case of activities more focused on exteroception, like moving an object in VR, the brain makes predictions mainly in direct response to external signals, e.g. related to the use of the VR controller and the outcomes seen in the VR scene. In the case of activities more focused on interoception, the external stimuli – the words on the page – are an input that activates embodied simulations, which in turn contribute to the emergence of cognitive and affective responses. In general, the two modes of embodied perception always work in combination, since interoception and emotions are always involved in the co-creation of our sensorimotor perceptions, and external stimuli continuously influence the exploration of internal body states ( Seth, 2014). However, when reading or imagining something, attention and intentions are more focused on interoception.

In addition, in many cases stimuli outside the focus of endogenous selective attention can influence conscious perception ( Schwitzgebel, 2007), also affecting the sense of presence. For instance, consider an athlete who is about to make a long jump at the Olympic Games and, through gestures, invites the crowd to cheer. During the performance, the athlete’s endogenous selective attention is very much focused on the movements needed for the run and jump. They experience a strong *core presence* (proximal intention to jump) and *extended presence* (distal intention to perform well at the Olympics). They are not consciously paying attention to the cheers of the crowd, but these certainly intervene in increasing their sense of presence and in helping them to find the right concentration. This is true also in the case of reading, when sensory selective attention is supposed to be completely directed on the page in front of the reader, in order to semantically process the sentences and make sense of the complexity of a narrative ( McNamara, 2007; Wolf, 2007). External stimuli unrelated to the reading activity can influence the narrative experience in various way ( Kuzmičová, 2016). For instance, if a reader is on the banks of a river while reading about a boat sailing at night and approaching a mysterious island, the peripheral perception of the sound of the water flowing beside them can increase their sense of presence, because it provides input data consistent with the predictive processing of the narrative, thus minimizing surprise and free-energy.

### 5.5 Social presence

The majority of the activities for which presence is mentioned somehow include other agents, be they real humans/animals, virtual avatars, or fictional characters. It has been found that observing an action performed by another individual – or its effects – activates the same brain areas as when an agent is directly performing such action, a phenomenon due to mirror neurons ( Rizzolatti *et al.*, 1996; Rizzolatti *et al.*,1998). Sensorimotor integration supported by the mirror matching system instantiates neural activations utilized not only to generate and control goal-related behaviours, but also to map the goals and purposes of others’ actions ( Barsalou, 2003; Gallese & Lakoff, 2005). This process establishes a direct link between one’s being and other beings: the observer uses their own resources to directly experience the world of the other by means of an unconscious process of motor resonance. This kind of embodied simulation with enaction of the other’s intentions also occurs when interacting with virtual avatars or reading about characters in a novel ( Gallese & Wojciehowski, 2011; Glenberg & Gallese, 2012).

Similarly to what happens with spatial presence, *social presence* is an evolutionary neuropsychological phenomenon related to predictive processing. On one side, an agent can recognize and simulate only intentions that they are able to enact ( Borroni *et al.*, 2011). On the other side, the correct enaction of such predictions reinforces their self-perception as a skilful agent capable of existing in relation to an environment which is continuously changing (spatial presence) and inhabited by others (social presence), as proposed by the free-energy principle. Through spatial presence, an agent controls their own action, comparing predictions with perceptual inputs to verify their successful enaction. Through social presence an agent recognizes and evaluates the actions and intentions of an other using the same predictive model. As for spatial presence, three different types of social presence emerged in time: *proto social presence*, the recognition that there is another self; *interactive social presence*, the acknowledgment that the intentions of the other can be directed towards the Self; *shared social presence*, a cognitive and affective matching of the other’s intentions ( Table 4).

**Table 4.** The types of social presence (reproduced, adapted, and expanded with permission from Riva, 2008).

Type	Relation with the Other	Recognized intentions	Social experience	Mediation (form/content)
Proto social presence	There is another Self (Other)	Motor intentions	Imitation	Perceptual
Interactive social presence	Intention of the Other is toward the Self	Proximal (Present) intentions	Interaction and communication	E.g. awareness of author’s narrative mediation; access to character’s speech and thoughts; interaction with character/avatar
Shared social presence	Self and Other share the same intention	Distal (Future) Intentions	Empathy and cooperation	Intellectually/emotionally significant content

Questionnaire items (cf. Table 2) inquiring about somebody’s co-location in space with another agent investigate proto social presence. Items about mind-reading and behavioural response to another agent are related to interactive social presence. And items about matching another agent’s emotions, feelings for another agent, connection with another agent, and understanding of another agent are related to shared social presence. The latter is often referred to with the name *empathy* and is considered particularly relevant for both VR experiences ( Herrera *et al.*, 2018; Martingano *et al.*, 2019) and reading fiction ( Burke *et al.*, 2016; Kuzmičová *et al.*, 2017). Sometimes, the term *identification* with the characters is also used ( Cohen, 2001).

### 5.6 Time and narrative

In the previous sections we described an agent’s self-perception in relation to a stimulus (spatial presence), the balance between the energy required by predictive processing and the agent’s explicit goals (flow), and the possible interaction with other agents (social presence). Let us now consider the role of time in such relationships, including the particular kind of temporal configuration of experience called “narrative”.

In psychology, narrative is often conceived in terms of its (mostly social) functions ( Bruner, 1986; Kidd & Castano, 2013; Mar & Oatley, 2008; Schank & Abelson, 1997), not with respect to the forms that afford such functions ( Pianzola, 2018) or the situated cognitive-affective response to narrative forms ( Hakemulder *et al.*, 2017; Kukkonen, 2019a; Kukkonen, 2020). Following this trend, a recent predictive processing account of narrative presents it as an exploration of possible meanings aimed at minimizing unexpectedness and uncertainty about the world and our actions in it: “narratives provide hypotheses that enable inference to the best prediction” ( Bouizegarene *et al.*, 2020). However, here we will focus on basic cognitive and affective processes at work *during* a narrative experience, processes which can serve various psychological functions. In brief, deep temporal models of the kind underlying narrative experiences allow an agent “to accumulate evidence *over different temporal scales* to find the best explanation for their sensations” ( Friston *et al.*, 2017, 388, emphasis added).

“On the active inference view, everything we do can be regarded as pursuing a narrative that resolves uncertainty” ( Friston *et al.*, 2020). However, we conceive narrative more specifically as a mode of cognition in which temporality is a *dominant factor* in the processing and organization of perception ( Pianzola, 2018; Walsh, 2018). Predictive processing is a time-sensitive process but not necessarily a narrative one, because predictions can be activated by intentions not motivated by the passage of time, namely movement in space. For instance, if an agent wants to reach a cup on the table, they have to make predictions and employ the available embodied simulation scripts: the goal is to extend arm and hand to grasp the cup and, to do it successfully, the agent has to minimize prediction errors about distance, which part of the cup to grasp, strength necessary to lift it from the table without spilling the coffee, etc. This is not a narrative process because temporality is less important than spatiality ^3^. In this perspective, it is more suitable to conceive of narrative as a scalar property of an experience, which can be more or less narrativized, i.e. more or less organized in a narrative way ( Abbott, 2014). Narrative organization is the cognitive articulation of a temporal sequence, that is, the relation an agent has with an environmental or internal stimulus whenever this is experienced (and interpreted) mainly with regard to its unfolding in time. In other words, the narrative sensory flow – the “story-driven experience” ( Caracciolo, 2014; cf. Pianzola, 2017) – emerges from the continuous interaction of perceptions and predictions ( Kukkonen, 2014), which involves the sensory design of the stimulus, the experiential background of the agent, the knowledge of media schemata, and the predictions based on these factors ( Kukkonen, 2020).

In general, the role of time in non-narrative experiences is simpler: the distorted perception of time investigated by many presence questionnaires is due to selective attention. When an agent feels present, time seems to flow more slowly because predictive processing is often focused on a very short time interval. For instance, when a tennis player feels a sense of presence during an intense set, their predictions are focused on hitting the ball (M- and P-intention) and scoring the point (D-intention), not on winning the match (broader D-intention). However, when defocusing because the experience is over, it can be surprising to observe that the sequence of short time intervals required to accomplish the goal set by the broader D-intention was longer than expected. This is because during the match the player did not update their predictions regarding the D-intention “winning the match”, since it was more urgent to make correct predictions for the M-, P-, and D-intentions necessary to hit the ball and score points. On the other hand, in the case of narrative experiences, sustained attention and predictions on a wider time range are required. For instance, to predict what a character would do, a reader needs to consider what they have read in the narrative up to that point, what information they received about the character’s past, what genre conventions are probably relevant (e.g. the murderer cannot be revealed in the first chapter; the first kiss comes after a suspense sustained for many chapters), how the plot developed so far, what are the probable character’s expectations, etc. This kind of predictive processing often requires skills that in everyday life are needed to make long-term decisions, thus used not as often as the skills required for other kinds of short- and mid-term predictions. Narrative deep temporal models ( Kukkonen, 2021) allow to practice how to accumulate evidence over different temporal scales to find the best explanation for perceptions.

Among the groups of items related to narrative absorption (cf. Table 2), those about the comprehension of narrative content are probably related to the widespread conception of narrative as a way to connect events in a meaningful and/or causal way ( Bruner, 1986; Bruner, 1991; Pier, 2008). In this light, the ability to understand how the events are linked would be a prerequisite for the emergence of narrative absorption. However, there are examples where such comprehension is not strictly necessary: the lack of comprehension about the interconnectedness of events and the absence of a closure that gives a stable meaning to the narrated events is often a characteristic of narratives ( Brooks, 1984), especially of complex ones ( Kiss & Willemsen, 2017). It is rather the continuous process of meaning-making during the unfolding of the narrative that usually creates a narrative tension that keeps the audience absorbed ( Baroni, 2007; Baroni, 2017; Sternberg, 1992). This aspect is investigated by the group of items related to suspense and anticipation.

One of the typical features of narrative is the generation of three main effects on the audience: suspense, curiosity, and surprise ( Sternberg, 1992; Sternberg, 2003). Suspense emerges when the fulfilment of a reader’s desire for information about a future outcome remains suspended because of an information gap. Conversely, curiosity emerges when the desire for information is about the past. And surprise emerges when new information reveals a prediction error and forces a reader to reconfigure their knowledge about something. Given that every narrative is unfolding in time towards an ending point, suspense is the most frequently occurring of the three dynamics, and this is probably the reason why it is the only one mentioned in the analyzed questionnaires. However, the three narrative effects influence presence in different ways, showing how narrative absorption is a phenomenon related to sustained attention, during which breaks in extended presence can occur without disrupting the absorbed state.

Suspense and curiosity tend to create a continuity between core and extended presence, whereas surprise often partially disrupts extended presence. In other words, with suspense and curiosity, the combination of the medium’s design and its content ( *syuzhet* and *fabula*, Tomaševskij, [1925] 1965) is designed to reduce prediction errors and keep sustained attention focused on the progression of the plot, thus creating an absorbing experience. In the case of surprise, the medium-content combination is designed to exploit prediction errors in order to create an experience that induces breaks in extended presence by attracting exogenous selective attention towards the narrative elements on which the wrong prediction was based and towards the new element that revealed the error (P-intention and narrow D-intention). However, at the same time, sustained attention is focused on the broader narrative context in which the prediction error occurred. Since narrative absorption is hierarchically higher than presence, less precision is assigned to errors coming from presence, which emerge at a lower level and can be minimized, through (epistemic) active inference ( Kukkonen, 2020; Pezzulo *et al.*, 2016), by reconfiguring knowledge about the story in light of the overall information provided by the narrative (broad D-intention). For instance, if the murderer is not whom the audience suspected to be, exogenous sensory selective attention shifts towards the disrupting element – requiring endogenous attention to work for a reconfiguration of the audience’s prediction about the plot (P- and narrow D-intention) – but sustained attention is still focused on finding out who the murderer is and on enjoying the narrative experience (broad D-intentions with higher precision). Other ways in which partial breaks in extended presence are achieved are: cognitive estrangement in science-fiction ( Suvin, 1979; Suvin, 2010), foregrounding of language due to a particular style ( Jacobs, 2015b; Jacobs & Willems, 2018; van Peer, 1986), hiding information to the audience, etc.

Narrative prediction errors can occur either with respect to the plot (e.g. a surprising event) or as difficulties in establishing precise predictions on the level of the narration (e.g. narrator who is unreliable, incomprehensible, etc.) ( Kukkonen, 2020). In both cases, prediction errors concern extended presence and the interoceptive states (distal tool) elicited by the narrative (proximal tool), but breaks in core presence can occur if there are prediction errors about language (proximal tool), like in the case of typos or grammatical errors. In such cases, the focus of attention shifts from the second-order mediation (plot) to the first-order mediation (use of grammar), inevitably creating a cognitive dissonance that results in an interruption of the absorbed state. More generally, literacy skills and the probability design of the narrative (e.g. progression of the story events as suggested in the narrative) work in combination in predictive processing. The probability design of the narrative unfolds through prediction errors that drive the narrative as plot events. These plot events are usually within a certain range of predictability (or precision), but there are also examples where readers get faced with prediction errors whose resonance is too ample and interrupts narrative absorption. Moreover, while reading is naturally paused at chapter breaks, ruptures at particularly striking and intense moments can be used to introduce breaks in the reading process of highly skilled readers to achieve aesthetic effects. However, in such cases it is probably more appropriate to talk about “aesthetic feelings” elicited by the narrative rather than about an absorbed state ( Kukkonen, 2020, pp. 89-90; cf. Jacobs, 2015b; Jacobs & Willems, 2018).

This PP theoretical model of narrative absorption is consistent with findings by ( Friston & colleagues, 2017; cf. Friston *et al.*, 2020), who run computational simulations to test similar cases of prediction errors related to the temporal processing of information. Word or lexical violations of expectations produced greater excursions of belief updating, but the pattern of neuronal encoding was the same as in the case of no violations. That is, more time was needed to make sense of the narrative because it was necessary to correct the model generating predictions about lexical use. Similarly, more processing time was required also in the case of semantic violations, for which the appearance of a certain word was more surprising than expected. The delay was due to the need to update the generative model with respect to the sentence context in which the word can appear. This kind of evidence does not say much about presence or narrative absorption – since it is still not clear what physiological signals can be reliably associated to such states ( Jacobs, 2015a; Jacobs & Lüdtke, 2017; Wiederhold *et al.*, 2001) – but it confirms that it is possible for our brain to complete a semantic-related task even if violations of the expected pattern occur. Similarly, it is plausible that sustained attention (and narrative absorption) can be maintained even if disturbed by local breaks in presence.

With its deep temporal models and the dynamics of anticipation (suspense), retrospection (curiosity), and recognition (surprise), narrative is a training ground for predictive processing, providing “alternate hypotheses that generalise and therefore preclude overfitting (sensory) data” ( Bouizegarene *et al.*, 2020). More specifically, in the case of fiction this happens on a safe ground, since brain regions devoted to emotion regulation tend to down-regulate subjective emotional intensity ( Mocaiber *et al.*, 2010; Sperduti *et al.*, 2017b; Sperduti *et al.*, 2017a).

## 6. Conclusions

We organized the knowledge produced in different fields of research working on concepts that describe a person’s experience when performing an engaging activity. Through a pragmatic investigation of the questionnaires used to measure these psychological states and a theoretical reconceptualization based on the predictive processing model of the mind, we suggest the following definitions:

*Spatial presence*: is an evolutionary neuropsychological phenomenon related to predictive processing. Agents are present where they are able to intuitively and successfully enact (i.e. without the involvement of reasoning) their implicit and explicit embodied predictions about possible action. When a prediction is not correctly enacted (break in presence), sensory selective attention is shifted towards the source of the prediction error, in order to facilitate the encoding of the wrong prediction in the brain, address it, and update the predictive model about the agent’s self-perceived agency.

*Social presence*: agents prereflexively recognize and evaluate the intentions and actions of others using the same predictive model that controls their own intention and actions. The others can be real, virtual, imagined, or fictional agents. When an agent successfully predicts and enacts the intentions of an other, social presence emerges, sometimes in the form of cognitive and affective matching of the other’s intentions.

*Narrative absorption*: when the temporal organization of perception is dominant for the enactment of predictions, agents undergo a narrative experience, which can be absorbing if a sense of presence emerges and sustained attention is kept on distal intentions. The deep temporal models underlying the narrative experience allow an agent to accumulate evidence over different temporal scales to find the best explanation for their sensations.

*Flow*: it is a characteristic of all three phenomena (spatial presence, social presence, and narrative absorption) related to the optimal balance between the skills possessed for an action and the challenges offered by the action. This corresponds to an optimal dynamic between predictions and prediction errors that does not disrupt the predictive model beyond an extent manageable by the agent only with moderate updates.

*Immersion*: we suggest avoiding the use of the noun in scientific research and prefer the adjective “immersive”, referring to the meaning commonly adopted in VR research, namely a quality referring to the technological features of the medium. In this sense, expression like “immersive medium”, “immersive experience”, or “immersive story” denote the potential of a medium or a specific mediated experience to elicit a sense of presence and narrative absorption. However, we are aware that in non-academic contexts the term “immersive” is commonly used with the meanings that we have here associated to presence and narrative absorption.

The described phenomena are based on the same brain dynamics, regardless of whether they are activated by a direct body stimulus or a stimulus mediated by physical, digital, or symbolic tools. The difference between the various stimuli, with respect to the emergence of presence, lies in a shift of sensory selective attention between exteroceptive and interoceptive perception and in the related attribution of higher/lower precision to predictions based on each of the two kinds of perception. The “book problem” and the “paradox of fiction” should be addressed as the attribution of higher precision to interoception, with the consequent regulation of interoceptive signals (emotions) by allocating more cognitive resources to their processing. Moreover, the interplay of intentions of different type (motor, proximal, distal intentions) also intervenes in the process, relativizing prediction errors and breaks in presence at lower levels.

To sum up, from the perspective of users, players, readers, and audience: an optimal balance between predictions and prediction errors (flow) facilitates the emergence of a sense of spatial presence, social presence, and narrative absorption. Alternatively, from the perspective of technology and media design: the more a medium or cultural artefact (e.g. narrative) is able to support the correct enactment of an individual’s predictions (spatial presence) and to clarify the intentions of others (social presence), the stronger is the sense of presence experienced with the medium or cultural artefact.

Our suggestions are helpful to shed light on theoretical confusion and guide the reconceptualization of the discussed phenomena, but, in order to empirically test the validity of our model, more thorough experiments should be conducted, combining self-reported measures with data from observation (e.g. Trasmundi & Cowley, 2020) and the tracking of neuro-physiological responses (e.g. Berta *et al.*, 2013; Jacobs *et al.*, 2016). Possible signals of presence or narrative absorption may be changes in heart rate, galvanic skin resistance, cerebral electrical activity, pupil dilations, eye movement, and facial expressions; but also measures related to task performance, such as reading speed or gestures’ fluidity in VR. For instance, by manipulating stimuli in order to influence interoceptive and exteroceptive predictions and prediction errors, we can test whether higher or lower level of spatial presence and narrative absorption are achieved, and how prediction errors of different precision can cause break in such subjective-phenomenal states. Moreover, using brain imaging we should be able to see an increased activation of the areas related to interoceptive (insula; Hassanpour *et al.*, 2016) or exteroceptive (temporal gyri spanning to the inferior occipital lobes; Salvato *et al.*, 2019) predictions. For a more detailed discussion about the efficacy of the various measures, see Paiva de Oliveira *et al.* (2016) and Jacobs (2015a).

To our knowledge, the model presented here is the first attempt at integrating results coming from a broad range of different fields and yielding in return a model that can be employed straightaway in such disciplinary fields. Indeed, it can be applied to understand the cognitive and aesthetic processing of narratives, inform the creation of media experiences, and develop more effective solutions for embodied medicine.

## Data availability

### Underlying data

OSF: Presence, flow, and narrative absorption questionnaires: a scoping review (supplementary material)

https://doi.org/10.17605/OSF.IO/RBZ8G ( Pianzola, 2021b)

This project contains the following underlying data:

- scoping_review_data_2021-02-26.xlsx (Human-readable version containing the 23 selected questionnaires with color coding of the items and summary model)
- scoping_review_data_2021-02-26.csv (Machine-readable version containing the 23 selected questionnaires with the respective annotations for each item)

Data are available under the terms of the Creative Commons Zero "No rights reserved" data waiver (CC0 1.0 Public domain dedication).
