# Supplementary material for: Presence, flow, and narrative absorption: an interdisciplinary theoretical exploration with a new spatiotemporal integrated model based on predictive processing
Source: Open Res Eur. 2021 Jul 23;1:28. Originally published 2021 Mar 26. [Version 2] doi: 10.12688/openreseurope.13193.2 (PMC10446082; doi:10.12688/openreseurope.13193.2)
Supplement: Peer review history

# Peer review: Reviewer response for version 2

**DOI:** 10.21956/openreseurope.15083.r27290

**Published:** 2021-08-04

**Authors:** Julian Kiverstein

**Affiliations:** 1 Amsterdam University Medical Centre, Amsterdam, The Netherlands

I do not have any additional comments. The authors clarified the main queries I had in response to their first draft in their reply. I am now happy to approve their submission for indexing.

If applicable, is the statistical analysis and its interpretation appropriate?

Yes

Is the study design appropriate and is the work technically sound?

Partly

Is the work clearly and accurately presented and does it engage with the current literature?

Yes

Are the conclusions drawn adequately supported by the results?

Yes

Are sufficient details of methods and analysis provided to allow replication by others?

Yes

Are all the source data and materials underlying the results available?

Yes

Reviewer Expertise:

Philosophy of mind and cognition.

I confirm that I have read this submission and believe that I have an appropriate level of expertise to confirm that it is of an acceptable scientific standard.

# Peer review: Reviewer response for version 1

**DOI:** 10.21956/openreseurope.14263.r26732

**Published:** 2021-05-04

**Authors:** Julian Kiverstein

**Affiliations:** 1 Amsterdam University Medical Centre, Amsterdam, The Netherlands

In this interesting and rich paper the authors show how the concept of ‘presence’ used in relation to virtual reality can be generalised to explain a reader’s experience of absorption in reading a novel. The paper uses results of questionnaires to offer a taxonomy of different concepts of presence that would allow for this generalisation. The author’s also show how to use the predictive processing framework to explain each of the concepts of presence they introduce. In my view the manuscript is near enough ready to be indexed but there are a few points I would find it useful to see further elaborated. In particular I did not find particularly plausible the claim that the withdrawal from reality into a fictive world can be accounted for in terms of increasing of precision on predictions of interoception, and downweighing of exteroceptive predictions. I agree that incoming sensory inputs are downweighed when we find ourselves absorbed by what we are reading but this seems more comparable to shift from task-dependent processing to spontaneous, stimulus-independent thought sometimes discussed in the mind-wandering literature. One of the authors of this paper has actually explored this idea in earlier work but it doesn’t receive much attention in this paper.

 The paper contains many ideas that sometimes read as digressions from the main themes of the paper. I could not see the necessity of the lengthy discussion of intention and the distinctions that are made between different types of intention in section 5. Part of the reason this is not as clear as it might have been is because the authors are very fast in their introduction of the concept of agency. They say this is needed to account for presence without explaining how and then quickly move on to providing a lengthy discussion of intention. I had similar reservations about the discussion of mediation – again the authors take for granted the connection to presence without spelling out how they see this.

**Smaller issues: **

1. In the definition of ‘presence’ what is meant by ‘fit’ – how do we fit in the real, virtual or fictional world?
2. Subjects are present, or they experience presence? (p.3, column 2)
3. Why would predictions relating to “symbolic tools” or “physical/digital tools” be attributed higher precision? This is too fast and needs unpacking.
4. Typo – “multiplicate” should perhaps be “multiply”?
5. Slater is referred to by their first name (p.4).
6. Is it necessary to describe place illusion in terms of “the qualia of having a sensation”? Not all researchers agree that qualia exist so this seems a rather theory-dependent way of describing the phenomenon (p.6).
7. I do not agree that the notion of perceptual presence as discussed by Noë and others is distinct from the concept of spatial presence as discussed later in this paper (p.6). Perhaps the authors take the difference to map onto the distinction between interoceptive and exteroceptive inference. But such a sharp distinction doesn’t in my view make sense given how interoception is at the core of all perception and action. This is a point that is emphasised in the work of Feldman-Barrett for example. Furthermore, presence is arguably tied to multisensory integration – the authors acknowledge this at various points (e.g. p.9). But multisensory integration is precisely the integration of exteroceptive, interoceptive and proprioceptive sensory states.
8. The PPMSC does not necessarily “downplay agency” (p.7) since counterfactuals are used to account for sensori *motor* contingencies. I guess it depends on what you mean by agency here but this criticism seems weak to me. The other points you make about genre conventions and personal relevance work better but then one wants to know how these factors are relevant for responding to the paradox of fiction/book problem.
9. Why does the book problem require a shift to interoception (p.8)? I would have thought motor and sensory imagery were just as important for experiencing presence in an imagined world. See point 8. I agree that precision is weighted towards the book as a source of sensory input and the imagery elicited by the text but why restrict this imagery to the responses of the internal viscera? Think for example of the example of Don Quixote riding his horse. The imagery here is not restricted the viscera and autonomic responses.
10.  What is meant by “evolutive tool”?
11. Possible typo: Your intention to move your hand is satisfied when you move your hand, not by your ability to move your hand (p.12).
12. It is unclear to me why the somewhat lengthy and elaborate discussion of intention in section 5.2 is included. I follow everything the authors write about intention in this section and its connection to motor schemas and propositional content but I don’t see how this material relates back to spatial presence. Perhaps the link is supposed to come from the discussion of peripersonal space but it is not clear to me how. I was following the argument more or less up until this section but the authors have now lost me. There are many concepts introduced in this paper and it is hard to keep track of everything.
13. How is the concept of mediation introduced in 5.3 related to presence?

The rest of the paper worked well. Look forward to reviewing a new version.

If applicable, is the statistical analysis and its interpretation appropriate?

Yes

Is the study design appropriate and is the work technically sound?

Partly

Is the work clearly and accurately presented and does it engage with the current literature?

Yes

Are the conclusions drawn adequately supported by the results?

Yes

Are sufficient details of methods and analysis provided to allow replication by others?

Yes

Are all the source data and materials underlying the results available?

Yes

Reviewer Expertise:

Philosophy of mind and cognition.

I confirm that I have read this submission and believe that I have an appropriate level of expertise to confirm that it is of an acceptable scientific standard, however I have significant reservations, as outlined above.

# Response

**Published:** 2021-07-20

**Authors:** Federico Pianzola

**Affiliations:** University of Milan Bicocca, Italy

Thank you very much for your comments, they will help us better express a few points of our argumentation.

 A. You said: “I did not find particularly plausible the claim that the withdrawal from reality into a fictive world can be accounted for in terms of increasing of precision on predictions of interoception, and downweighing of exteroceptive predictions”. 

 This statement includes two elements: (1) how can a written text achieve the experience of presence in readers (the so-called book problem) and (2) why would we seek it out?  We argue that in real-world environments, interoception is limited (through sensory attenuation) so that we can perceive, move and explore in exteroception with ease. Inversely, by limiting exteroception in reading (through the poor stimulus of written language), we can place greater attention on interoception and explore inner states (see Kukkonen 2020; 2019). This greater explorative freedom in interoception accounts also for the attraction of reading literature. When we decide to read/watch fiction, we do so because we are mostly interested in the effects it has on us and we take pleasure in having emotions and thoughts prompted by a narrative. That is, we engage in this activity – and in the predictive processes involved in it – with the distal intention of receiving some kind of interoceptive perception. Whereas, when we interact with some object, person, or animal, our predictions are more sensitive to our perception of these external entities and to their behavior in response to our interaction with them. Accordingly, our intentions are ultimately focused on achieving a non-equilibrium state (free energy minimization) in relation to our existence in a space inhabited by other objects and agents. When reading/watching fiction, our internal states are more important than making correct sensorimotor predictions about the real space in which we are or the fictional space we are observing/imagining. Consider the case of a video game of a flight simulator: it is not uncommon for players to intentionally crash the plane they are flying into a mountain or some other object, just because it is fun to see the explosion resulting from this action. We might follow a character doomed to die in a fictional narrative for comparable reasons. In this case, like in many other forms of interactions with virtual and fictional worlds, the interoceptive states prompted by our actions are more relevant (have a higher precision) than the resulting situation in terms of specific changes happening to objects and space: it is enough to roughly predict that the plane is going to explode, without predicting how its shape or that of the mountain with which it collided will be modified. For this reason, in Figure 2b we differentiate between intentions *mainly *directed towards different entities. Interoception is more salient when reading/watching fiction, but predictions regarding space and other agents are very important, too. That is why we consider spatial presence and social presence as phenomena that contribute to the emergence of narrative absorption. We are aware that another article is needed to better present all the nuances of our possible engagement with fiction, and to clarify to what extent spatial and social presence are necessary for narrative absorption to emerge. We are going to write that article soon. In the meantime, we look forward to continuing the discussion.

 A final point that we want to underline, is that the focus on interoception does not downplay the critical role played by motor and sensory imagery. We agree with you: they are important for experiencing presence in an imagined world because their content is used to generate the interoceptive prediction. In simpler words, motor and sensory imagery generate an interoceptive prediction – based on the individual’s previous experiences - that is then compared with internal interoceptive signals. The individual feels present when predictions and signals do not differ. You raise the related issue of spontaneous cognition / mind-wandering in relation to absorption in reading. The article mentioned by you (Fabry and Kukkonen 2019) proposes that literary texts depend on elements of concentration to create the necessary absorption, but that within such absorption, readers engage in mental time travel within the fictional world and establish self-relevance by relating events they read about to their own memories spontaneously in mind-wandering. The relation between these two dimensions, and the textual cues provoking them, however, still need to be developed in greater detail. One of us is currently preparing a paper detailing an inventory of embodied cues for mind-wandering and attention in reading. Following what we said before, a possibility is that absorption is related to the possibility of the literary text of generating an interoceptive prediction, a possibility that also depends on the previous experience of the individual and on its ability to give meaning to the text.  

 B. With respect to your concern about the many digressions found in the article, we agree that the paper is quite long, but we felt that it was necessary to introduce the different types of intentions and mediations. As we tried to clarify in point A above, the distinction between proximal and distal intentions is necessary to understand how precision weighting works in relation to fiction. Moreover, the same distinction is useful to account for how different media and technologies can affect spatial presence, social presence, and narrative absorption. Namely, as we discuss in the examples in sections 5.3 (Mediated action) and 5.6 (Narrative and time), the dynamics involving focused attention and the different types of intentions can explain situations that are very common when reading/watching fiction or interacting with virtual worlds. The many possible configurations regarding a medium, its design in conveying a certain content, the type of content presented, and the cultural expectations that agents have are not something that can occasionally occur, they are what makes each work of fiction, game, and art having cognitive-affective effects on us. Therefore, a theory of narrative, fiction, and technological mediation more broadly, should account for how these various aspects influence perception and to what extent they are interdependent. Anyway, we will try to sharpen some parts of section 5, also considering your useful suggestion about the concept of agency and the relation between presence and mediation.

 Regarding the smaller issues you mentioned:

*1.   In the definition of ‘presence’ what is meant by ‘fit’ – how do we fit in the real, virtual or fictional world?*

 With the verb “fit” we refer to the pursuit of an optimal interaction with the environment (including other agents), i.e. the outcome of successful predictive processing.

 2. *Subjects are present, or they experience presence? (p.3, column 2)*.

 We use the two phrases as synonyms: we are present when we experience presence.

 3. *Why would predictions relating to “symbolic tools” or “physical/digital tools” be attributed higher precision? This is too fast and needs unpacking.*

 Actually, we claim that “The main difference is related to the *precision *… attributed to the different layers of embodied predictions (Kukkonen, 2019a; Seth & Friston, 2016): predictions related to physical/digital tools and physical/digital objects attribute higher precision to exteroceptive and proprioceptive layers and/or vestibular information, according to the activity performed (section 5.3). Instead, predictions related to symbolic tools attribute higher precision to interoceptive information (section 5.4)”.

  6. *Is it necessary to describe place illusion in terms of “the qualia of having a sensation”? Not all researchers agree that qualia exist so this seems a rather theory-dependent way of describing the phenomenon (p.6)*.

 We agree that it is not necessary, here we are reporting Slater’s account.

 7. *I do not agree that the notion of perceptual presence as discussed by Noë and others is distinct from the concept of spatial presence as discussed later in this paper (p.6). Perhaps the authors take the difference to map onto the distinction between interoceptive and exteroceptive inference. But such a sharp distinction doesn’t in my view make sense given how interoception is at the core of all perception and action. This is a point that is emphasised in the work of Feldman-Barrett for example. Furthermore, presence is arguably tied to multisensory integration – the authors acknowledge this at various points (e.g. p.9). But multisensory integration is precisely the integration of exteroceptive, interoceptive and proprioceptive sensory states*.

 We think they are very different concepts: “perceptual presence” is a phenomenological property attributed to objects/agents in relation to how they are perceived by someone, whereas “spatial presence” is a subjective-phenomenal state qualifying the experience of the perceiving agent. It may be possible that perceptual presence is required for the emergence of spatial (and social) presence, but this is a philosophical discussion that goes beyond the scope of our article. We agree with the position of Feldman-Barrett (2017): “allostasis (predictively regulating the internal milieu) and interoception (representing the internal milieu) are at the anatomical and functional core of the nervous system. These insights offer a range of new hypotheses—e.g. that reappraisal and other regulation processes are accomplished with predictions that categorize sensory inputs and control action with concepts”. In our view, presence is based on allostatic processes that are focused either on exteroception or interoception.

 8. *The PPMSC does not necessarily “downplay agency” (p.7) since counterfactuals are used to account for sensorimotor contingencies. I guess it depends on what you mean by agency here but this criticism seems weak to me. The other points you make about genre conventions and personal relevance work better but then one wants to know how these factors are relevant for responding to the paradox of fiction/book problem*.

 We could have expressed this idea better. The main point is that Seth’s Predictive Processing SensoryMotor Contingencies model (PPSMC) presents representations as counterfactually poor, in comparison to “real world” action, because they can provide only a limited amount of sensory data to generate counterfactuals. In this conception, the assumption is that exteroceptive stimuli are more important for the generation of counterfactuals, whereas we claim that intentions and interoception can be as important, if not more, than exteroception, and thus generate a rich array of counterfactual predictions, which can make fictive, represented situations “look real”.  With respect to genre conventions and personal relevance, we will elaborate a bit more in the revision but will devote a follow-up article to exploring their role in more detail.  

 9. *Why does the book problem require a shift to interoception (p.8)? I would have thought motor and sensory imagery were just as important for experiencing presence in an imagined world. See point 8. I agree that precision is weighted towards the book as a source of sensory input and the imagery elicited by the text but why restrict this imagery to the responses of the internal viscera? Think for example of the example of Don Quixote riding his horse. The imagery here is not restricted the viscera and autonomic responses*. 

 We talked about a “shift in the precision of embodied predictions towards *interoception*”. Sensorimotor perception is important, too, when reading/watching fiction (the mediation of a symbolic tool like a book extends our peripersonal space, enabling us to enact sensorimotor actions performed by fictional agents), but the relevance of interoception is usually higher, namely with respect to distal intentions (see point A above). We also agree that imagery is important for experiencing presence in an imagined world because its content is used to generate interoceptive predictions. 

 10.** *What is meant by “evolutive tool”?*

  In this context, we meant a cognitive tool that significantly contributed to our evolution as a species, in terms of both our sensory coupling with the environment and our social and cultural relations with other agents. In particular, we suggest that by moving towards more advanced levels of presence the individual develops a more complex self and is able to enact more complex intentions.

 13 *. How is the concept of mediation introduced in 5.3 related to presence?*

 The mediation of technology modifies the extension of our peripersonal space, thus enabling us to have a broader range of sensorimotor perceptions related to fiction or virtual spaces/agents. This extension then allows us to feel spatial presence prompted by perceptions had by other (fictional) agents. We will clarify our argumentation in the revised article.   References Fabry, R. E., & Kukkonen, K. (2019). Reconsidering the Mind-Wandering Reader: Predictive Processing, Probability Designs, and Enculturation. *Frontiers in Psychology*, *9*, 2648. https://doi.org/10.3389/fpsyg.2018.02648 Feldman Barrett, L. (2017). *How Emotions Are Made: The Secret Life of the Brain*. Houghton Mifflin Harcourt. Kukkonen, K. (2019). Exploring Inner Perceptions. Interoception, Literature, and Mindfulness. *Journal of Consciousness Studies*, *26*(11–12), 107–132. Kukkonen, K. (2020). *Probability Designs: Literature and Predictive Processing*. Oxford University Press.

# Peer review: Reviewer response for version 1

**DOI:** 10.21956/openreseurope.14263.r26663

**Published:** 2021-04-06

**Authors:** Karl J. Friston

**Affiliations:** 1 Wellcome Centre for Human Neuroimaging, UCL Queen Square Institute of Neurology, London, UK

I enjoyed reading this theoretical review and account of presence, flow and narrative absorption. I thought that the authors did an excellent job in bringing together the attending conceptual and theoretical themes. I will comment from the point of view of active inference and predictive processing in theoretical neurobiology.

 My main recommendations would be to clarify the relationship between active inference and predictive processing – and to leverage a couple of formal issues in active inference that substantiate the authors treatment. In particular, perhaps you could consider the following:

 I thought it was very useful to frame the discussion in terms of narratives. The notion of a narrative is key for the kind of deep generative models that you refer in Section 5. As you note, narratives have both a spatial and temporal aspect; the latter being probably more definitive for narratives. It might be useful to make this explicit with a sentence like the following in the introduction:

 “Much of what follows rest upon the notion of a narrative that has an inherently sequential, ordinal or temporal aspect that—as we will see later—generally entails a deep temporal structure. ”

 In terms of the relationship between active inference and predictive processing, this can be a little subtle. From your perspective, I think you can assume they are the same thing. This means you might want to nuance a couple of references to active inference. Specifically, at the bottom of page 3, you refer to an extension of the standard predictive processing scheme that incorporates interoceptive information. In fact there is no standard predictive processing scheme. What you are referring to is generally called interoceptive inference, which is a special case of active inference.

 Could you delete "and active inference models" on page 7: you seem to imply that active inference does not include counterfactual probabilities. In fact, the policy selection that underwrites active inference is a selection from counterfactual policies. You intimate this in your footnote 2, where you refer to "a form of enactive inference". This is just active inference.

 In section 5, I was compelled by your treatment of narratives and anticipation. I think it would be useful to bring out the central role of generative models and, implicitly, shared narratives in terms of narrative absorption. Perhaps with something like:

 “The key construct for predictive processing (i.e., active inference) is the notion of a generative model. In other words, everything we do in the service of securing evidence for our model of how sensations are generated; the other words, a model of the latent causes or states generating the sensorium. In this setting, a narrative becomes part of a deep generative model that, usually, entails a separation of temporal scales, where things unfold more slowly with hierarchical depth. An obvious example here is language; however, any narrative could be cast as the ordered sequence of contexts that specify a subordinate sequence events that unfold for the duration of any context at the level above.

 The notion of narrative-as-a-generative model becomes especially prescient in the current setting because we are generally seeking narratives that best explain the sensory evidence at hand. If this sensory evidence is generated by a (possibly virtual) other, then inferring the narrative that they have in mind can be an extremely ill-posed problem. However, this problem is finessed if you and I share the same narrative (i.e., generative model). In other words, if we share a narrative, I can use my generative model of my own engagement with the world to predict what you will do next; thereby enabling me to infer your intentions from your actions. This has been cast in terms of solving (or perhaps dissolving) the problem of neuronal hermeneutics—that underlies mutual understanding and theory of mind. Put simply, to identify with a character in a story, I just need to infer that the character shares my narratives, intentional stances and prior beliefs (i.e., my generative model)."

 There are a few simulations that speak to the implicit synchronization you refer to under this formulation. For example, (Friston and Frith, 2015 ^1^). This may offer a formal basis for the notion of a shared social presence?

 There is quite a lot of work on artificial curiosity that may be useful for your treatment of narrative absorption. This ranges from intrinsic motivation in robotics to curiosity and fun in generalized artificial intelligence (Oudeyer and Kaplan, 2007 ^2^; Parr and Friston, 2019 ^3^; Schmidhuber, 2010 ^4^). In the setting of active inference, curiosity is the thing that underlies epistemic affordance and would be essential for simulating "narrative absorption". The most explicit simulation of this is in terms of the aha moment (Friston *et al*., 2017 ^5^). Although simulated in terms of abstract rule recognition, one might imagine exactly the same principles apply to the dénouement of a story or narrative.

 Minor points

 At the bottom of page 4, you refer to Mel Slater as "Mel".

 On page 4, the phrase "it does not multiplicate entities" was a bit confusing. What does multiplicate mean?

 I hope that these comments help in any revision.

If applicable, is the statistical analysis and its interpretation appropriate?

Yes

Is the study design appropriate and is the work technically sound?

Yes

Is the work clearly and accurately presented and does it engage with the current literature?

Yes

Are the conclusions drawn adequately supported by the results?

Yes

Are sufficient details of methods and analysis provided to allow replication by others?

Yes

Are all the source data and materials underlying the results available?

Yes

Reviewer Expertise:

Theoretical neurobiology.

I confirm that I have read this submission and believe that I have an appropriate level of expertise to confirm that it is of an acceptable scientific standard.

## References

[1] : A Duet for one. *Conscious Cogn* .2015;36: 10.1016/j.concog.2014.12.003 390-405 10.1016/j.concog.2014.12.003 25563935PMC4553904

[2] : What is Intrinsic Motivation? A Typology of Computational Approaches. *Front Neurorobot* .2007;1: 10.3389/neuro.12.006.2007 6 10.3389/neuro.12.006.2007 18958277PMC2533589

[3] : Generalised free energy and active inference. *Biol Cybern* .113(5-6) : 10.1007/s00422-019-00805-w 495-513 10.1007/s00422-019-00805-w 31562544PMC6848054

[4] : Formal Theory of Creativity, Fun, and Intrinsic Motivation (1990-2010). *IEEE Transactions on Autonomous Mental Development.* .2010;2(3) : 10.1109/TAMD.2010.2056368 230-247 10.1109/TAMD.2010.2056368

[5] : Active Inference, Curiosity and Insight. *Neural Comput* .29(10) : 10.1162/neco_a_00999 2633-2683 10.1162/neco_a_00999 28777724

# Response

**Published:** 2021-07-20

**Authors:** Federico Pianzola

**Affiliations:** University of Milan Bicocca, Italy

Thank you for your insightful comments, we will take them into account in revising our article. Here we would like to reply to a few of them.

 1. With respect to the relationship between active inference and predictive processing, we agree that it is out of the scope of our article to distinguish between various theories of predictive processing, so we will nuance our references to the active inference approach.

 2. With respect to our discussion of narrative, we think it is useful to make a distinction between different uses of the term "narrative". If narrative is something like "narrative explanation" of behaviour or event sequences, then, yes, we think it can be conceived as a generative model. This would be a cognitively basic notion of narrative. However, if narrative is something like "fictional narrative" or "literary narrative", then it refers to an artificial structure that works with or against generative models and that explores possibility spaces outlined by basic narrative scripts. A predictive processing model can cover both, but we suppose you are referring only to the first kind of "narrative".

 2a. You mentioned the case in which, “if we share a narrative, I can use my generative model of my own engagement with the world to predict what you will do next”. In this sense, “narrative” is a broader cultural model functioning as high-level module in PP. However, we do not think it is useful to conceive of all generative models as narratives. It depends on the intentions of the predictive process: if the temporal dimension is dominant (e.g. taking up a cup that you put on the table is part of the cognitive script of ordering a coffee in a café and waiting for it to be ready), then it is useful to talk about narrative processing, a narrative organization of perceptions. But if the temporal unfolding of an action is not very relevant, then it is inaccurate to talk about “narrative”, e.g. if two of us are in the café and got our coffee, and you put your cup on the table where we are sitting, I do not consider my visual processing of your action as a narrative, because in this situation I am normally not expecting (predicting) me to do any action with your coffee. I can infer from your action that you have no intention of drinking coffee for some time, but I think it is not appropriate to conceive of this inference as a narrative. It would be a different case if you explicitly invited me to try the new special blend you ordered, in which case your invitation will prompt a shared narrative about trying something new, hoping it tastes good, and me giving you an opinion about it. Again, predictions have a narrative form only in some cases, namely when its temporal organization is dominant in our processing of perceptions.

 2b. You also mention that “to identify with a character in a story, I just need to infer that the character shares my narratives, intentional stances and prior beliefs (i.e., my generative model)”. We do not think this is always the case. Like all the other mentioned subjective-phenomenal states (presence, social presence, flow, and narrative absorption), identification with a character — extended social presence, in the terms we used in the article — can occur with different degrees of intensity and can be achieved through many different processes. What you suggest is a situation in which identification is probably quite strong, but it can also happen that we identify with a character even though we do not share with them any specific intentional stance or prior belief. For instance, it may be the case that our prior beliefs only align with the narrative plot more broadly, like when reading a “rags to riches” story and relating it to our life narrative of personal success. In this case, we are able to identify with the character in a broad sense, even though, let’s say, the character’s success is due to luck (or the author’s fiat) only. An identification might occur, because readers wish a similar outcome for themselves, even though they cannot expect as much luck but have to achieve the results through hard work. In other cases, it is even more simple, identification can be prompted even by the mention of a physical trait – or other small details – shared by reader and character, as one of us noticed it is frequently happening for young readers (Pianzola et al., 2020).

 3. Yes, the model proposed in Friston and Frith (2015) can be a formal basis for the notion of social presence, and it can also explain other aspects of reading fiction. For instance, a familiarity with the genre “detective fiction” can be conceived as a form of shared precision expectations between author and reader. Knowing that it is likely that a character will be murdered is a piece of shared information that influences the reader’s prediction about the “sensations generated by another” through a text. One of us articulated in more detail such larger, genre-based predictions in literary reading processes (Kukkonen, 2020).

 4. Thanks for pointing us to research on artificial curiosity. Modelling curiosity in artificial agents is a very interesting topic and is very relevant with respect to how "artificial environments" such as VR, games, and narrative can be formally modelled reducing the complexity of the subjective-phenomenal state that they generate, including narrative absorption. It would be interesting also to simulate and compare how artificial agents process narrative differently than humans. Namely, as artificial agents are not embodied (but our model is), it needs to be asked whether a level of embodiment also would have to be modelled for the artificial agent. Another important aspect to be considered in creating a computational model of narrative processing is that “artificial curiosity” seems to be a term including all the three processes we have presented – curiosity, surprise, and suspense – but their dynamics are different both in temporal and cognitive terms, as we discuss in the article.   References Kukkonen, K. (2020). *Probability Designs: Literature and Predictive Processing*. Oxford University Press. Pianzola, F., Rebora, S., & Lauer, G. (2020). Wattpad as a resource for literary studies. Quantitative and qualitative examples of the importance of digital social reading and readers’ comments in the margins. *PLoS ONE*, *15*(1). https://doi.org/10.1371/journal.pone.0226708
